# Evaluation of single-sample network inference methods for precision oncology

**DOI:** 10.1038/s41540-024-00340-w

**Published:** 2024-02-15

**Authors:** Joke Deschildre, Boris Vandemoortele, Jens Uwe Loers, Katleen De Preter, Vanessa Vermeirssen

**Affiliations:** 1 Lab for Computational Biology, Integromics and Gene Regulation (CBIGR), Cancer Research Institute Ghent (CRIG), Ghent, Belgium; 2 Department of Biomedical Molecular Biology, Ghent University, Ghent, Belgium; 3 Department of Biomolecular Medicine, Ghent University, Ghent, Belgium; 4 Lab of Translational Onco-genomics and Bio-informatics, Center for Medical Biotechnology (VIB-UGent), Cancer Research Institute Ghent (CRIG), Ghent, Belgium

**Keywords:** Computational biology and bioinformatics, Cancer, Biochemical networks

## Abstract

A major challenge in precision oncology is to detect targetable cancer vulnerabilities in individual patients. Modeling high-throughput omics data in biological networks allows identifying key molecules and processes of tumorigenesis. Traditionally, network inference methods rely on many samples to contain sufficient information for learning, resulting in aggregate networks. However, to implement patient-tailored approaches in precision oncology, we need to interpret omics data at the level of individual patients. Several single-sample network inference methods have been developed that infer biological networks for an individual sample from bulk RNA-seq data. However, only a limited comparison of these methods has been made and many methods rely on ‘normal tissue’ samples as reference, which are not always available. Here, we conducted an evaluation of the single-sample network inference methods SSN, LIONESS, SWEET, iENA, CSN and SSPGI using transcriptomic profiles of lung and brain cancer cell lines from the CCLE database. The methods constructed functional gene networks with distinct network characteristics. Hub gene analyses revealed different degrees of subtype-specificity across methods. Single-sample networks were able to distinguish between tumor subtypes, as exemplified by node strength clustering, enrichment of known subtype-specific driver genes among hubs and differential node strength. We also showed that single-sample networks correlated better to other omics data from the same cell line as compared to aggregate networks. We conclude that single-sample network inference methods can reflect sample-specific biology when ‘normal tissue’ samples are absent and we point out peculiarities of each method.

## Introduction

In order to understand the complex molecular interactions at play in tumor pathogenesis, high-throughput omics data have been generated at an increasing pace^1^. Modeling these data in biological networks allows for determining the key molecules and processes that drive tumorigenesis^2,3^. Traditionally, network inference methods rely on many samples to contribute sufficient information to the learning process and to counteract the curse of dimensionality in the omics data, i.e. the number of genes by far outnumbering the number of samples. Methods to accomplish this on tissue level from bulk omics data are already well-established, and rely on varying underlying statistical and mathematical principles such as correlation, mutual information, Bayesian networks and regression^4–6^. We and others have shown that different computational methods reveal complementary aspects of the ‘true’ underlying networks^4,7–9^. However, these methods infer networks based on numerous samples and therefore determine a general estimate of gene interactions largely shared by that group of samples. Hence, they result in population-level networks, averaging the phenotypic effects of individual patients or samples. For clinical applications, we need to be able to interpret and extract meaningful information from omics data of a single individual to be able to direct individualized treatment in precision medicine^10^.

Currently, several approaches are being explored to analyze omics data from a single sample or patient, and are referred to in literature as single-sample, single-subject, sample-specific, patient-specific and personalized methodologies. Deep n-of-1 phenotyping, where multiple omics are profiled in a single individual at different locations in the body longitudinally, is envisioned to be essential for the early detection and personalized treatment of cancers^11^. Obtaining multiple samples from one patient is nonetheless not straightforward due to an increased cost, increased surgical risk, or limited tumor size. Moreover, single-sample or patient-specific networks can be built from single cell RNA-seq data of a single subject, where the profiling of many cells inherently contains the variability required to infer the statistical dependencies between genes^12^. However, single cell omics data have specific limitations such as high-dimensionality, sparsity and overdispersion and network inference methods are still being optimized to deal with these issues. Also, single cell technologies are currently more expensive and hardly implemented in the clinic as compared to bulk protocols. On bulk transcriptome data, several methods extract relevant biological knowledge from individual samples without requiring a large disease cohort, as reviewed in^13^. They either provide a gene-centric view on differentially expressed (DE) genes or a pathway-centric view on deregulated pathways, comparing a single sample against a reference cohort or a control sample^13–16^. In addition, VIPER can predict protein activity from regulon enrichment on single-sample gene expression signatures obtained using a reference set^17^. The single-sample Network Perturbation Assessment (ssNPA) is a method for subtyping samples based on single-sample deregulation of their gene networks^18^. While these methods allow for biological interpretation of omics data at the individual level, they do not generate biological networks or gene interactions for single samples or patients.

To address this, single-sample network inference methods have been developed that can infer a biological network for a single sample from bulk RNA-seq data. Several of these methods make use of an aggregate network constructed from all samples and a statistical wrapper to infer single-sample features within these networks. Others devise a specific statistic to directly obtain single-sample networks. Optionally, networks can be pruned by a background network, such as a protein-protein interaction network. The Single-Sample Network (SSN) algorithm calculates the significant differential network between the Pearson Correlation Coefficient (PCC) networks of a set of reference samples on the one hand and that same reference set plus the sample of interest on the other hand, both using the STRING database as background network^19^. The authors experimentally validated that SSN identified functional driver genes contributing to resistance in non-small cell lung cancer cell lines. Subsequently, SSN has been applied to breast and colon cancer to study stage- and subtype-related networks and to identify diagnostic and prognostic biomarkers^20,21^. LIONESS also uses a leave-one-out approach in aggregate network inference to come to a single-sample network, and through linear interpolation incorporates information on both the similarities and the differences between the networks with and without the sample of interest^22^. LIONESS has the major advantage that any network inference method of choice can be used to construct the aggregate networks, and has been applied e.g. to study sex-linked differences in colon cancer drug metabolism^23^. The Individual-specific Edge-Network Analysis (iENA) algorithm constructs single-sample PCC node-networks and single-sample higher-order PCC edge-networks by altered PCC calculations of the expression data of the sample of interest and a set of reference samples^24^. On the other hand, Sample Specific Perturbation of Gene Interactions (SSPGI) computes individual edge-perturbations based on differences between the rank of genes within the expression matrix of normal samples and individual samples of interest^25^. The Cell-Specific Network construction (CSN) method transforms the expression data into more stable, statistical gene associations, rendering a binary network output at single cell or single-sample resolution, for single or bulk RNA-seq data respectively^26^. The recent method SWEET also consists of linear interpolation like LIONESS, but integrates genome-wide sample-to-sample correlations to weigh subpopulation sample sizes that can cause network size bias^27^. Whereas SSN, SSPGI and CSN only apply a differential approach, LIONESS, iENA and SWEET also take into account commonalities between single-sample and aggregate networks.

The above mentioned single-sample network inference methods have mainly been applied by the research groups that developed them and a systemic neutral comparison is still missing. Only limited comparisons have been performed, which either focused on a limited number of methods, focused on downstream network control methods or made use of metabolomics data with a limited number of features^22,28–30^. Furthermore, many of the compared methods rely on ‘normal tissue’ reference samples to contrast the tumor samples, which might not be available for all tumor types or in all precision oncology cases. The CCLE database offers multiple omics, including transcriptomics, on a large panel of comprehensively characterized human cancer cell lines and thus represents an ideal playground to apply and compare single-sample network inference methods^31,32^. In this study we constructed single-sample coexpression networks using SSN, LIONESS, SWEET, iENA, CSN and SSPGI for lung cancer and brain cancer samples. We found that each method constructed networks with distinct topologies at the level of edge weight distributions and network characteristics. The node strengths of the different single-sample networks tended to cluster together according to tumor subtype. For both lung and brain samples, we identified the largest part of subtype-specific hubs in SSN, followed by LIONESS and iENA networks. Hubs in these single-sample networks also differed the most from hubs in the aggregate network. However, for all methods, hubs displayed enrichment for subtype-specific IntOGen/COSMIC drivers for NSCLC and glioblastoma, the two largest sample groups in respectively lung and brain samples. Differential node strengths between tumor subtypes were mainly detected in SSN, LIONESS and SSPGI networks. Yet, differentially strong nodes were not enriched for known subtype specific driver genes. In SSN, LIONESS and iENA, we noticed a tendency for lower node strengths for the bigger subtype sample group in both lung and brain samples, while this potential bias was absent in SWEET, CSN and SSPGI. Finally, we showed that single-sample networks correlated better to other omics data from the same cell line as compared to aggregate networks. Single-sample networks from SSN, LIONESS and SWEET resulted in the largest average correlation coefficients, for both lung and brain samples, and for proteomics and copy number variation data. Overall, we conclude that single-sample networks in the absence of ‘normal tissue’ samples were able to reflect sample-specific information better than aggregate networks and that different tools have their peculiarities that should be taken into account.

## Results

### Subtype-specific gene expression in lung and brain CCLE cell lines

**Fig. 1:**
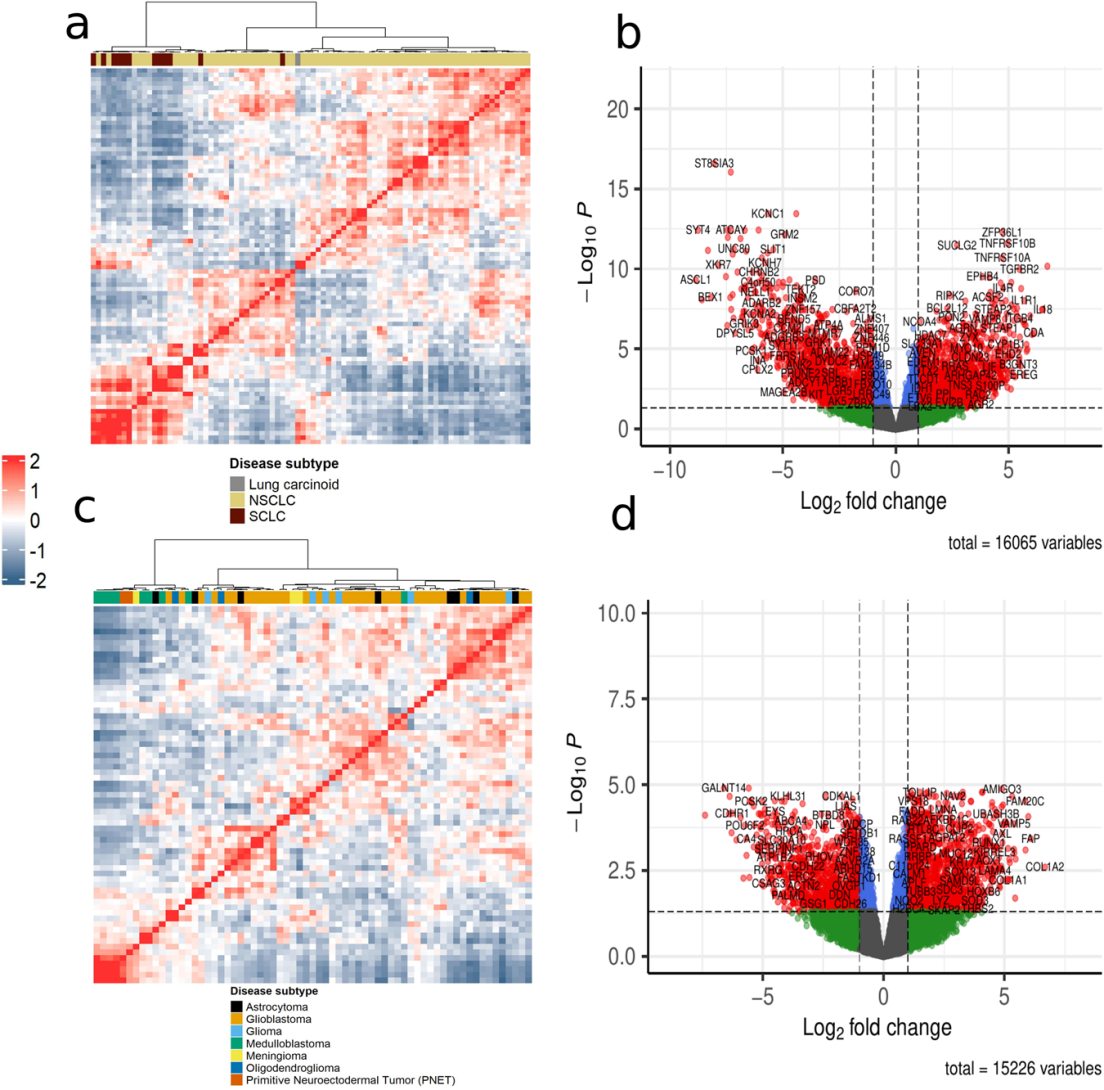
Lung and brain cancer cell lines exhibit extensive transcriptional differences between subtypes. **a** Heatmap showing hierarchical clustering of Z-scores of pairwise Spearman correlations of gene expression in lung samples using Ward’s linkage. **b** Volcano plot of DE genes in NSCLC versus SCLC. Significantly DE genes are colored in red (p-adj <= 0.05 & |LFC | > 1, moderated t-test). **c** Heatmap showing hierarchical clustering of Z-scores of pairwise Spearman correlations of gene expression in brain samples using Ward’s linkage. **d** Volcano plot of DE genes in glioblastoma versus medulloblastoma. Significantly DE genes are colored in red. (DE differentially expressed, NSCLC non-small cell lung carcinoma, SCLC small-cell lung carcinoma, p-adj adjusted *p* value, LFC log fold change).

In order to evaluate single-sample network inference methods in the absence of healthy control reference samples, we set out to compare SSN, LIONESS, SWEET, iENA, CSN and SSPGI on gene expression profiles from CCLE lung and brain cancer cell lines^31^. We identified cell lines that closely matched their corresponding tumor tissue with regard to gene expression, and retained 86 lung and 67 brain cancer cell lines (Methods)^33^. These are further split into subtypes including 73 non-small cell lung carcinoma (NSCLC), 12 small cell lung carcinoma (SCLC), 1 lung carcinoid, 36 glioblastoma, 9 astrocytoma, 8 glioma, 9 medulloblastoma, 3 meningioma, 3 oligodendroglioma and 2 primitive neuroectodermal tumor (PNET) cell lines (Methods). An initial clustering of lung expression profiles showed that all but one of SCLC samples clustered separately from NSCLC samples (Fig. 1a). We further compared gene expression in both cancer subtypes and identified 1510 up- and 1553 downregulated genes in NSCLC versus SCLC samples (absolute log fold change (abs(LFC)) >= 1, adjusted p-value (padj) <= 0.05) (Fig. 1b). A clustering of brain expression profiles revealed one subcluster containing all but one of medulloblastoma and all PNET samples (Fig. 1c). Due to limited sample sizes for meningioma, oligodendroglioma, PNET and glioma, we choose to perform subsequent differential analyses in brain between glioblastoma and medulloblastoma samples. In total, 1354 and 1043 genes were up- and downregulated in glioblastoma versus medulloblastoma samples (Fig. 1d). In the supplementary information, we extended some analyses to other tumor subtypes (brain) or sub-subtypes (lung) (see further). Hence, we detected substantial transcriptional differences between tumor subtypes for both lung and brain samples.

### Construction of single-sample networks

**Fig. 2:**
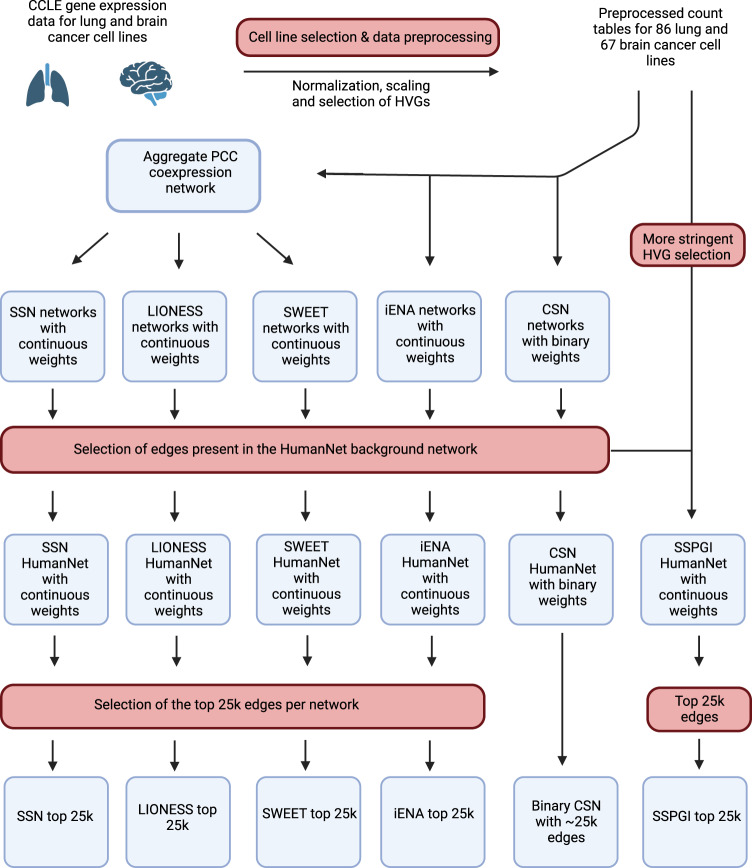
Overview of single-sample network construction and network pruning. Expression data of lung and brain cancer cell lines were downloaded from CCLE, after which samples were selected and data preprocessed (Methods). Aggregate coexpression networks were constructed for both tissues using Pearson’s correlation (PCC), and single-sample networks using SSN, LIONESS, SWEET, iENA, CSN and SSPGI. SSPGI was not scalable and was therefore run with a lower number of highly variable genes (HVGs) as compared to the other methods. SSPGI inherently already used HumanNet as background network, while the other single-sample networks were pruned for edges present in the HumanNet network. Next, the top 25k edges were selected in SSN, LIONESS, SWEET, iENA and SSPGI networks, whereas all ~25k edges were selected in the binary CSN networks.

For both tumor types, we selected highly-variable genes (HVG) for functional gene network construction. First, we inferred an aggregate, undirected coexpression network using PCC, representing all samples. Next, single-sample networks were inferred using LIONESS, SSN, SWEET, iENA, CSN and SSPGI. We slightly modified several tools to run with PCC as the underlying network inference method and in absence of ‘normal tissue’ reference samples (Methods, GitHub). The choice of PCC as underlying network inference approach allowed for a consistent comparison between single-sample networks, as some methods exclusively function with PCC, and between the single-sample and the aggregate networks. We further pruned the aggregate and single-sample networks by selecting edges present in the HumanNet network, an integrated human functional gene network that was used as background network (Methods, Fig. 2)^34^. This resulting lung aggregate and single-sample functional gene networks consisted of 5454 nodes and 53 296 edges, covering respectively 30.50% of proteins and 10.14% of HumanNet interactions. Due to lack of scalability, lung SSPGI networks were slightly smaller and comprised 4814 nodes connected by 43 193 edges. Due to gene ID conversion based on a more recent genome annotation, lung SWEET networks were slightly larger comprising 5706 nodes connected by 55 806 edges (Methods, Supplementary Table 1). The aggregate brain network and single-sample brain networks constructed using SSN, LIONESS, CSN and iENA comprised 4741 nodes and 42 948 edges after pruning for HumanNet interactions, while 4686 nodes and 42 206 edges remained in the SSPGI networks. Again, SWEET networks for brain samples were slightly larger comprising 4936 nodes and 45 724 edges.

### Different single-sample network inference methods generate distinct network topologies

First, we aimed to explore the network topology of the aggregate and single-sample networks^35^. Supplementary Fig. 1 depicts the distribution of edge weights in the aggregate networks, as well as across all edges in single-sample networks. Also, it shows the distribution of the average weight of each edge individually across all samples. For lung samples, edge weights in SSN networks ranged between [-0.3, 0.35], while edge weights in SWEET and LIONESS networks ranged between [-1.5, 1.5] and [-25, 30] respectively. iENA lung networks had an edge weight distribution similar to those constructed by LIONESS, with weights ranging between [-25, 32]. CSN produced networks with binary weights of either zero or one, such that all edges present in the network of a specific sample had a weight of exactly one. Finally, networks constructed by SSPGI had edge weights in the interval [-15000, 15000], with non-continuous values since it is a rank-based method. We observed similar edge weight distributions in networks constructed for brain samples. Interestingly, edge weights in SWEET networks followed a distribution highly similar to their respective aggregate network, while for other methods there is a clear deviation. In both tissues, networks constructed by SSN, LIONESS and iENA were predominantly characterized by edge weights close to zero. Due to the binary nature of edge weights in CSN networks, either zero or one, a significant proportion of edges within each single-sample CSN network was associated with weight zero and thus absent in the network. On average, these networks contained 27 814 and 24 399 edges for lung and brain samples, respectively. We therefore selected the top 25 000 edges in SSN, LIONESS, SWEET, iENA and SSPGI networks, rendering networks comparable in size across all methods. These networks, which had previously already been pruned by HumanNet, are further referred to as top 25k networks. The signs of edge weights in these networks were mostly consistent between methods for SSN, LIONESS and iENA. SSPGI and SWEET edges showed some inconsistencies with other methods, while CSN edge weights are binary and thus incomparable (Supplementary Fig. 2).

The top 25k networks varied in edge weight distributions as well as network topology (Supplementary Tables 1–3)^36^. The aggregate networks had an order of magnitude more connected components than single-sample networks, and also displayed higher clustering coefficients and lower node and edge betweenness. Thus, aggregate networks were more tightly connected with shorter paths between nodes. Overall, topological differences between single-sample networks themselves were rather small, with SPPGI having a lower clustering coefficient than the rest for both tumor types. Thus, although constructed on the same data as the aggregate networks and subjected to similar edge selection procedures, each single-sample network inference method built distinct single-sample networks that were mostly different from the aggregate network, both at the level of edge weight distribution and network topology.

### Exploration of single-sample networks

**Fig. 3:**
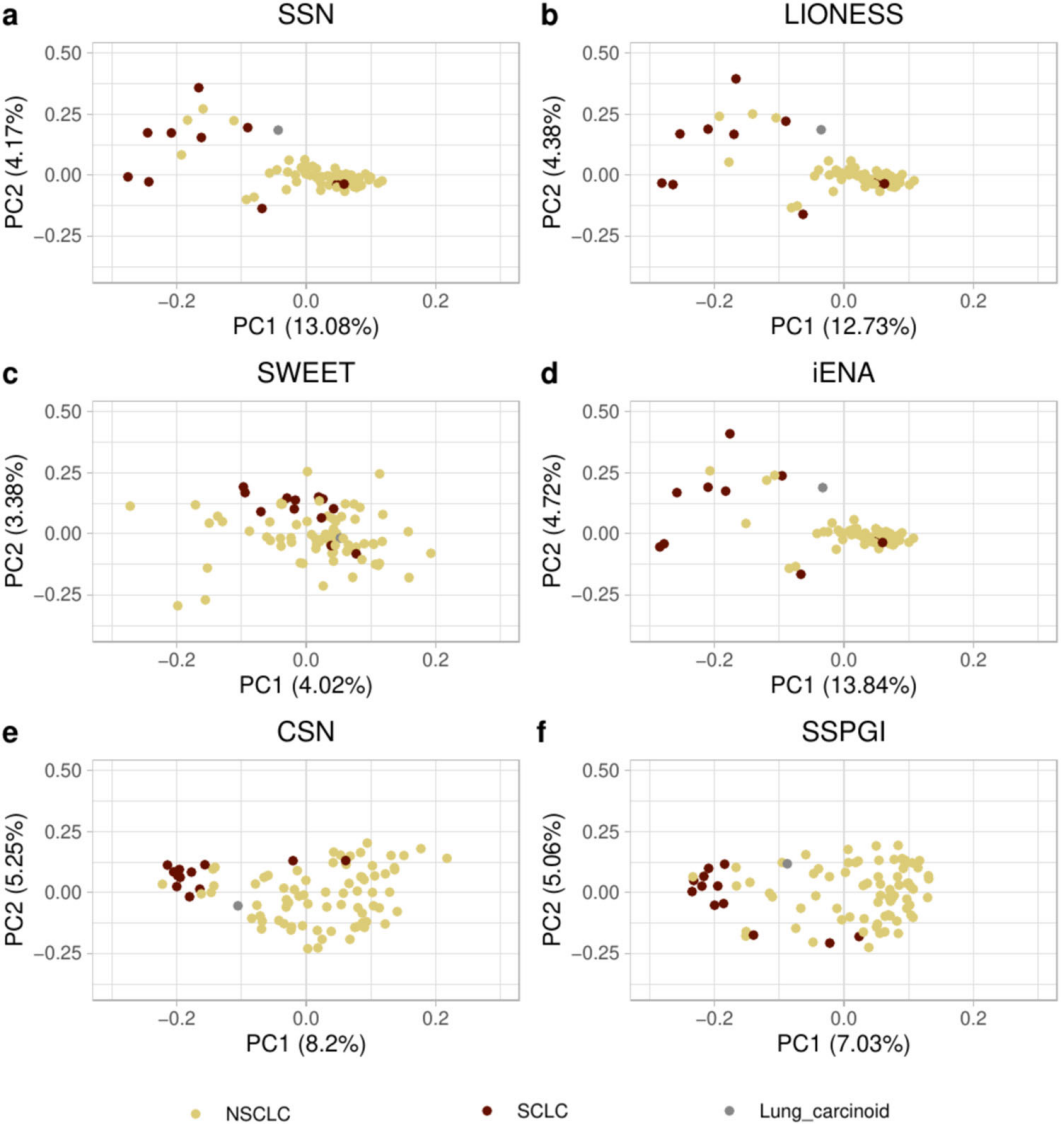
Visualization of lung samples after projecting the node strengths i.e. the sum of absolute edge weights of the single-sample networks onto their first two principal components. We performed PCA analysis on top 25k lung networks constructed using (**a**) SSN, (**b**) LIONESS, (**c**) SWEET, (**d**) iENA, (**e**) CSN and (**f**) SSPGI. Each dot represents one single-sample network constructed from a cell line corresponding to a given cancer subtype. (NSCLC non-small cell lung carcinoma, SCLC small cell lung carcinoma, PCA principal component analysis).

Next, we inquired to what extent LIONESS, SSN, SWEET, iENA, CSN and SSPGI provide relevant biological insights at the sample-specific and subtype-specific level. Therefore, we first calculated the node strengths^37^ i.e. the sum of absolute edge weights for each node in the single-sample networks, and projected these onto their first two principal components using Principal Component Analysis (PCA). For both lung and brain top 25k networks, the different single-sample networks tended to cluster together according to subtype, but only up to 18% for lung and up to 28% for brain of the total variance is being explained by the first two PCs, with decreasing values going from iENA, SSN, LIONESS, CSN, SSPGI to SWEET (Fig. 3, Supplementary Fig. 2, Supplementary Fig. 3).

### Analysis of hubs in single-sample networks

**Fig. 4:**
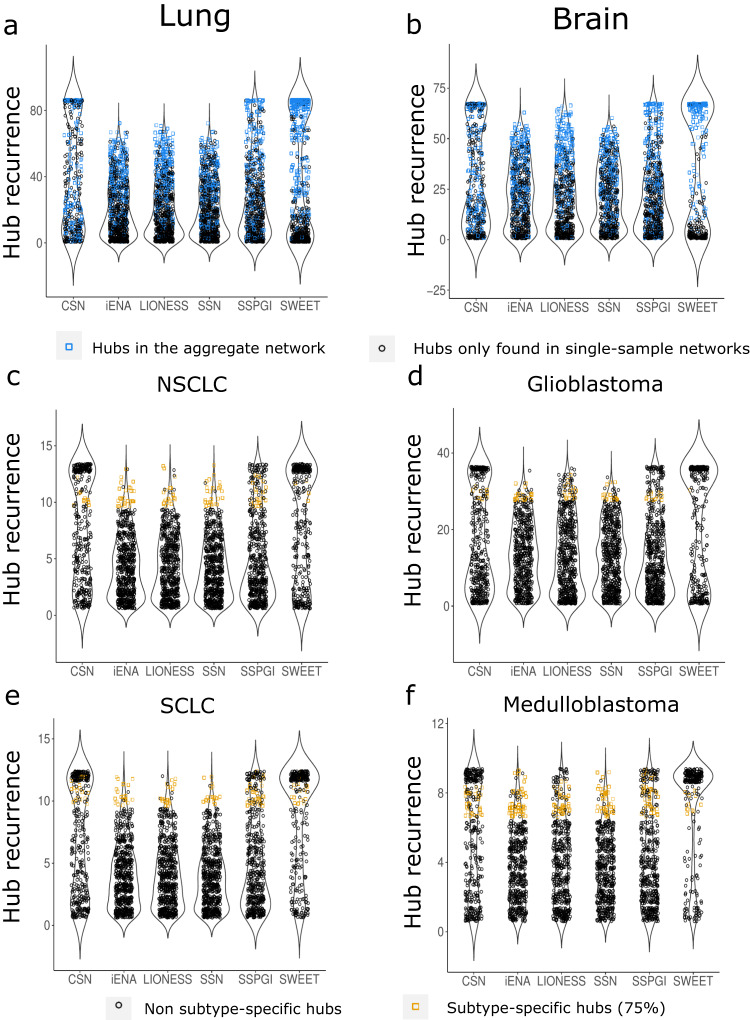
Hub recurrence in single-sample networks. Each dot represents one gene identified as hub, and the y-axis shows how many times this hub recurs in a sample group. **a**, **b** Plots containing all hubs over all single-sample networks per inference method for lung and brain respectively. Hubs overlapping with the 200 hubs retrieved from the aggregate network of the same tissue are colored in blue. **c**–**f** Plots containing all hubs over all single-sample networks of one subtype per inference method for lung NSCLC (non-small cell lung cancer) and SCLC (small cell lung cancer), brain glioblastoma and medulloblastoma respectively. For each subtype, the subtype-specific hubs are colored in yellow and defined as the hubs (i) occurring in at least 75% of the networks of that subtype and (ii) the hubs selected in (i) that were not overlapping with the selected hubs of the other subtype for the same tissue.

We further identified hubs by selecting the top 200 most connected nodes in each single-sample network and aggregate networks (Methods). As single-sample network inference methods are designed to capture heterogeneity between samples of a tumor type, we expect to some extent different hubs in different samples, and ideally these hub genes are related to the cancer subtype of a given sample. To test this, we first assessed the recurrence of hub genes (i.e. the number of times a given gene is identified as hub across a group of samples) in networks constructed using a given method for lung (Fig. 4a) and brain samples (Fig. 4b). All methods constructed single-sample networks with the majority of hubs being unique to only one or a few samples, for both brain and lung. However, SWEET, CSN and SSPGI networks showed hubs recurring in all samples: respectively 110, 96 and 18 hub genes overlapped between all lung samples and 131, 76 and 21 hub genes overlapped between all brain samples. Some hub genes were regularly recurring within SSN, LIONESS and iENA networks, but none overlapped across all samples. Furthermore, the top 200 hub genes of the aggregate networks of lung and brain were consistently recurring among the hub gene sets identified in single-sample networks, and this was most obvious in CSN and SWEET networks (Fig. 4a, b). Together, these observations suggest that SSN, LIONESS and iENA produced networks that were inherently more different from each other than SWEET, CSN or SSPGI networks. Also, hubs in the aggregate networks tended to be hubs in the single-sample networks.

Hub genes should ideally be related to the cancer subtype of a given sample, and thus similar hubs should be found within sample groups. We thus grouped all NSCLC, SCLC, glioblastoma and medulloblastoma single-sample networks and evaluated the union and intersection of hub gene sets within these groups (Supplementary Tables 4 and 5). CSN and SWEET represented with the lowest number for the union of hub gene sets within sample groups, indicating a poorer hub diversity over single-sample networks within a tumor-specific or tumor subtype-specific sample group. Especially for SSN, iENA and LIONESS there is limited overlap in hubs e.g. for NSCLC, the largest sample group, there were zero hubs in common across all samples. In CSN and SWEET networks on the other hand, close to or more than 100 hubs were overlapping in any given sample group, indicating a highly similar network topology. Nonetheless, all single-sample networks did have regularly recurring hubs per cancer subtype group (occurring in at least 75% of the samples in a given group). We next identified subtype-specific recurring hubs as those hubs that regularly recur within one sample group, and do not overlap with regularly recurring hub genes in other sample groups (Fig. 4c–f). On Fig. 4, each dot represent one gene that was identified as a hub, and the y-axis represents the number of times that given hub is found across a given sample group. The highest proportion of subtype-specific versus non-subtype-specific hubs among highly-recurring hubs was observed for SSN networks, followed by iENA and LIONESS. Moreover, these methods generated a lower amount of highly recurring hubs. SWEET, CSN and SSPGI had more recurring hub genes that were less specific to the cancer subtype of a given sample group (Fig. 4c–f). Similar results were obtained upon investigating more subtypes (brain) or sub-subtypes (lung) (Supplementary Fig. 5).

**Fig. 5:**
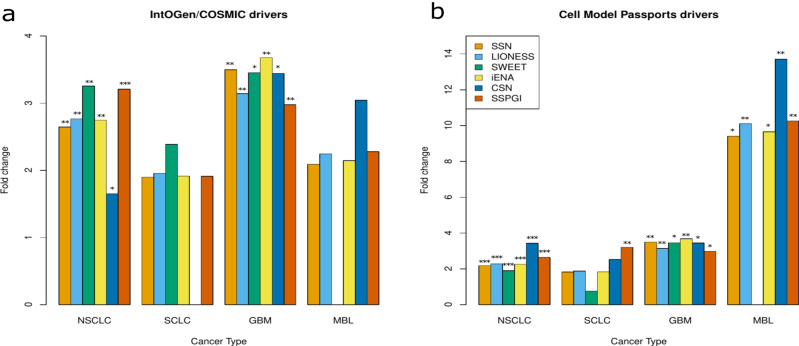
Enrichment of known subtype-specific cancer driver genes in hub gene sets. **a** IntOGen/COSMIC subtype-specific drivers. **b** Cell Model Passports drivers for the specific CCLE cell lines. The top 200 most connected nodes in each single sample network were identified as hub genes. Hubs genes were then grouped per sample type and enrichment for known subtype specific cancer driver genes was assessed (*p < 0.05, **p < 0.01, ***p < 0.001, hypergeometric test). NSCLC non-small cell lung cancer, SCLC small cell lung cancer, GBM glioblastoma, MBL medulloblastoma.

Next, we assessed whether these hub lists were enriched for known cancer driver genes. We downloaded a list of known drivers from IntOGen and COSMIC/Cancer Gene Census for NSCLC, SCLC, glioblastoma and medulloblastoma, and additional cancer subtypes (analyses in supplementary information), as well as CCLE cell line specific cancer drivers from the Cell Model Passports database, and assessed their presence in the sets of hubs per sample group. For both databases, we observed in addition to some overlap, many tumor subtype-specific and sub-subtype-specific cancer driver genes (Supplementary Fig. 6). Out of 69 and 59 driver genes for NSCLC and SCLC from IntOGen/COSMIC respectively, 23 were present in the aggregate HumanNet lung network. On the other hand, NSCLC and SCLC samples were characterized by 213 driver genes in total according to Cell Model Passports, of which 74 were present in the aggregate HumanNet lung network. Known IntOGen/COSMIC drivers for medulloblastoma and glioblastoma respectively comprised 57 and 45 genes, of which 9 and 16 respectively were present in the aggregate HumanNet brain network. These samples were further characterized by 72 driver genes according to Cell Model Passports, while 19 of these were present in the aggregate brain network. After concatenating hubs per sample group, we found that each method constructed networks in which hub genes were enriched for subtype-specific IntOGen/COSMIC drivers for NSCLC and glioblastoma, the two largest sample groups. Cell Model Passport drivers on the other hand were enriched in hub genes identified in NSCLC, glioblastoma and medulloblastoma samples (Fig. 5a, b). An analysis of additional sample groups, defined by cancer sub-subtype (lung) or subtype (brain), confirmed that each tool is capable of prioritizing subtype-specific genes as hubs, although with decreasing sample numbers it became more difficult to observe this (Supplementary Fig. 7, Table 1). There was no single tool that clearly outperformed the others.

**Table 1:** Overlap between hubs identified per sample group and known subtype-specific cancer driver genes from IntOGen/COSMIC and Cell Model Passports

	*SSN*	*LIONESS*	*SWEET*	*iENA*	*CSN*	*SSPGI*
SCLC (*n* = 12)	*ERBB4*, *EGFR*, *SOX2*, *NOTCH1*, *FGFR2*, TGFBR2, PDGFRA, PTPRB, ESR1, HOXA11	*ERBB4*, *EGFR*, *SOX2*, *NOTCH1*, *FGFR2*, TGFBR2, PDGFRA, PTPRB, ESR1, HOXA11	*ERBB4*, *EGFR*, *NOTCH1*, TGFBR2	*ERBB4*, *EGFR*, *SOX2*, *NOTCH1*, *FGFR2*, TGFBR2, PDGFRA, PTPRB, ESR1, HOXA11	TGFBR2, PTPRB, ESR1, HOXA11	*EGFR*, *NOTCH1*, *PTPN13*, *FGFR2*, TGFBR2, PDGFRA, PTPRB, ESR1, HOXA11, CDKN2A, AR, ESR1
NSCLC (*n* = 73)	*ERBB4*, *SOX2*, *KDR*, *PTPN13*, *CDKN2A*, *FGFR2*, *EGFR*, *SLC34A2*, FBN2, LEF1, *NOTCH1*, TGFBR2, MET, FGFR4, COL1A1, EBF1, CDKN2A, PTPRB, CDH10, NFATC2, CDH11, ELN, CHD1, FN1	*ERBB4*, *SOX2*, *KDR*, *PTPN13*, *CDKN2A*, *FGFR2*, *EGFR*, *SLC34A2*, FBN2, LEF1, *NOTCH1*, TGFBR2, MET, FGFR4, COL1A1, EBF1, CDKN2A, PTPRB, CDH10, NFATC2, CDH11, ELN, CHD1, FN1	*ERBB4*, *EGFR*, *SOX2*, GRIN2A, SLC34A2, FBN2, NOTCH1, TFGBR2, FGFR4, EBF1, CDH11	*ERBB4*, *SOX2*, *KDR*, *PTPN13*, *CDKN2A*, *FGFR2*, EGFR, *SLC34A2*, F FBN2, LEF1, *NOTCH1*, TGFBR2, MET, FGFR4, COL1A1, EBF1, CDKN2A, PTPRB, CDH10, NFATC2, CDH11, ELN, CHD1, FN1	*EGFR*, *FGFR2*, GRIN2A, EGFR, FBN2, *NOTCH1*, TGFBR2, MET, FGFR4, COL1A1, EBF1, PTPRB, CDH10, NFATC2, CDH11, ELN, CDH1, FN1	*ERBB4*, *SOX2*, *KDR*, *PTPN13*, *CDKN2A*, *FGFR2*, *EGFR*, *SLC34A2*, FBN2, LEF1, *NOTCH1*, TGFBR2, MET, FGFR4, COL1A1, EBF1, CDKN2A, PTPRB, CDH10, NFATC2, CDH11, ELN, CHD1, FN1
NSCLC_Adeno (*n* = 45)	*NOTCH1*, *PTPN13*, *FGFR2*, *EGFR*, *SLC43A2*, FBN2, LEF1, TGFBR2, MET, COL1A1, EBF1, CDKN2A, PTPRB, CDH10, ELN, FN1	*NOTCH1*, *PTPN13*, *FGFR2*, *EGFR*, *SLC43A2*, FBN2, LEF1, TGFBR2, MET, COL1A1, EBF1, CDKN2A, PTPRB, CDH10, ELN, FN1	*EGFR*, *SLC34A2*, *CTNND2*, CDKN2A, NFATC2, CDH1	*NOTCH1*, *PTPN13*, *FGFR2*, *EGFR*, *SLC43A2*, FBN2, LEF1, TGFBR2, MET, COL1A1, EBF1, CDKN2A, PTPRB, CDH10, ELN, FN1	*EGFR*, *NOTCH1*, *MET*, *CDH10*, *EGFR*, FBN2, TGFBR2, MET, COL1A1, EBF1, PTPRB, CDH10, ELN, FN1	*NOTCH1*, *PTPN13*, *FGFR2*, *EGFR*, *SLC43A2*, FBN2, LEF1, TGFBR2, MET, COL1A1, EBF1, CDKN2A, PTPRB, CDH10, ELN, FN1
NSCLC_Squamous (*n* = 13)	*EGFR*, *FGFR3*, *PDGFRA*, *EBF1*, *CDH10*, *CTNND2*, GRIN2A, FBN2, *CDKN2A*, NFATC2, CDH1	*EGFR*, *FGFR3*, *PDGFRA*, *EBF1*, *CDH10*, *CTNND2*, GRIN2A, FBN2, *CDKN2A*, NFATC2, CDH1	*EGFR*, *NOTCH1*, *LATS2*, *EBF1*, *CTNND2*, GRIN2A	*EGFR*, *FGFR3*, *PDGFRA*, *EBF1*, *CDH10*, *CTNND2*, GRIN2A, FBN2, *CDKN2A*, NFATC2, CDH1	*EGFR*, *NOTCH1*, *MET*, *CDH10*, GRIN2A, FBN2, NFATC2, CDH1	GRIN2A, FBN2, CDKN2A, NFATC2, CDH1
NSCLC_Large_cell (*n* = 6)	FGFR4	FGFR4	*/*	FGFR4	FGFR4	FGFR4
Glioblastoma (*n* = 12)	*CACNA1D*, *PDGFRA*, *FGFR4*, *CDKN2A*, *COL1A1*, *EGFR*, MET, WNK2, PTPRB, *TP53*	*CACNA1D*, *PDGFRA*, *FGFR4*, *COL1A1*, *EGFR*, MET, WNK2, PTPRB, *TP53*	*EGFR*, *PDGFRA*, *COL1A1*, *TP53*, MET, WNK2, PRPRB	*CACNA1D*, *PDGFRA*, *FGFR4*, *CDKN2A*, *COL1A1*, *EGFR*, MET, WNK2, PTPRB, *TP53*	*CACNA1D*, *PDGFRA*, *COL1A1*, *EGFR*, MET, WNK2, PTPRB, *TP53*	*CACNA1D*, *PDGFRA*, *FGFR4*, *COL1A1*, *EGFR*, MET, WNK2, PTPRB, *TP53*
Medulloblastoma (*n* = 36)	*WNK4*, *TP53*, *GRIN2A*	*WNK4*, *TP53*, *GRIN2A*	*WNK4*, *TP53*, GRIN2A	*WNK4*, *TP53*, *GRIN2A*	*WNK4*, *TP53*, *GRIN2A*	*WNK4*, *TP53*, *GRIN2A*
Astrocytoma (*n* = 8)	TP53	TP53	/	TP53	TP53	TP53
Glioma (*n* = 6)	EGFR, TP53	EGFR, TP53	EGFR	EGFR, TP53	EGFR, TP53	EGFR, TP53

The top 200 most connected nodes in each single-sample network were selected as hub genes. These were then grouped per sample group (SCLC, NSCLC, medulloblastoma or glioblastoma), and the intersection with known subtype-specific driver genes from IntOGen/COSMIC (italic), Cell Model Passports (underlined) or both (italic underlined) was determined.

Overall, we found that SSN, LIONESS, SWEET, iENA, CSN and SSPGI construct single-sample networks, in which different genes were identified as hubs for different samples. For a given sample, the average overlap of hub genes across methods was 25 genes in lung networks, and 42 genes in brain networks. Further, we observed varying degrees of subtype-specificity within hub genes, with CSN and SWEET networks having the lowest diversity among hub genes. Furthermore, we found a significant enrichment of NSCLC and glioblastoma driver genes from both IntOGen/COSMIC and Cell Model Passports for all methods.

### Differential node strength in single-sample networks

**Fig. 6:**
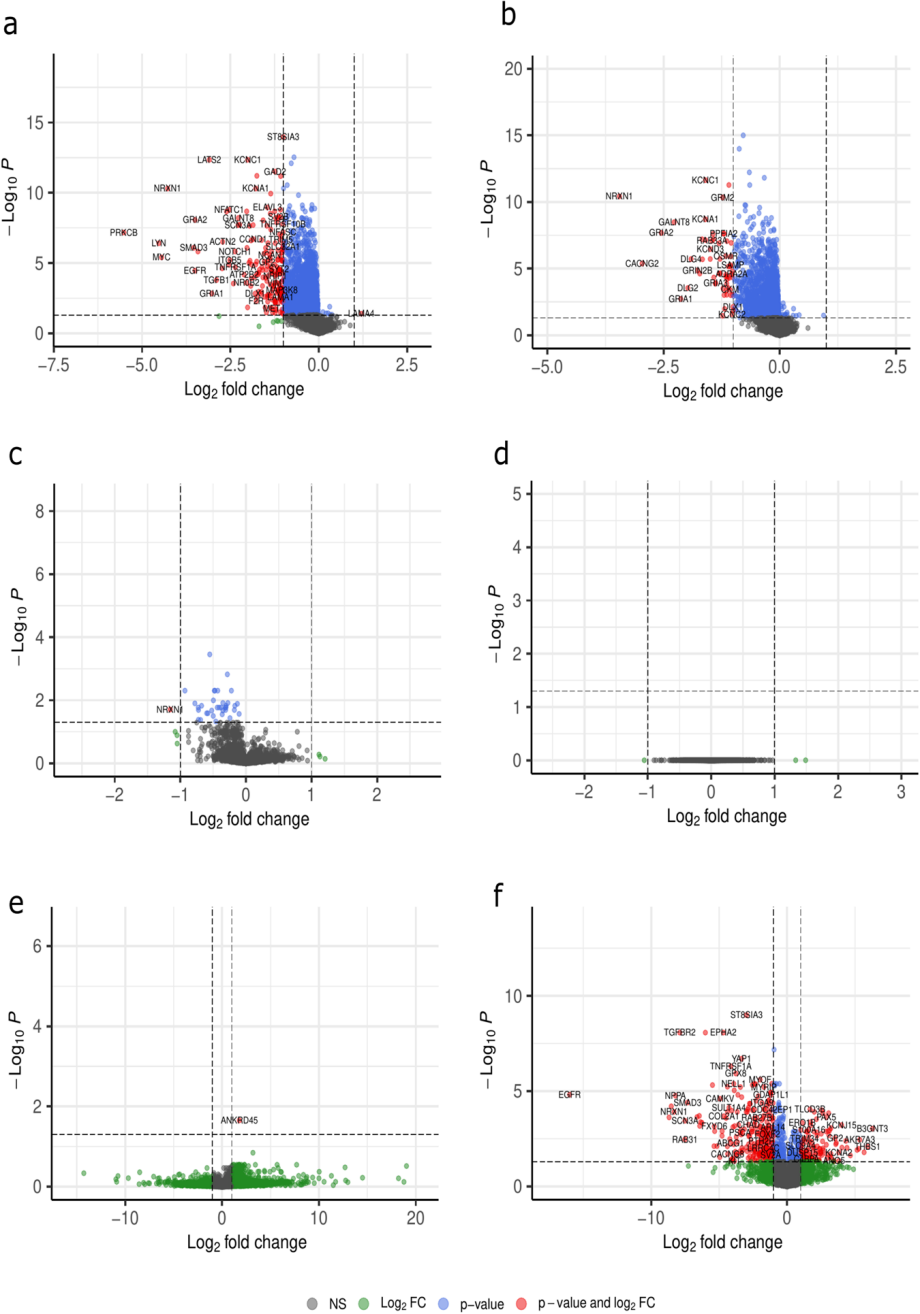
Single-sample networks display distinct differential node strength between non-small cell lung carcinoma (NSCLC) vs small cell lung carcinoma (SCLC) across network types. The node strength, or sum of absolute edge weights, was calculated for all nodes in top 25k networks constructed by (**a**) SSN, (**b**) LIONESS, (**c**) SWEET, (**d**) iENA, (**e**) CSN and (**f**) SSPGI. Differentially strong nodes (p-adj < 0.05 & |LFC | >= 1, moderated t-test) in NSCLC versus SCLC (LFC < 0 means lower in NSCLC than SCLC) were identified using linear modeling and an empirical Bayes procedure. (p-adj adjusted *p* value; LFC log fold change, NS non-significant).

The node strength quantifies how strongly a node is directly connected to other nodes in the network^37^ i.e. by summing all absolute weights of edges connected to the given node. In the undirected single-sample networks we calculated the node strength of a given node as the sum of absolute edge weights of that node, after scaling weights to values between -1 and 1. Using linear modeling and an empirical Bayes procedure^38^, we identified differentially strong nodes (p-adj < 0.05 & |LFC | > 1) between NSCLC and SCLC samples: 59 in LIONESS, 192 in SSN and 363 in SSPGI networks. Only one node was significantly differentially strong in CSN and SWEET lung networks, and zero in iENA networks (Fig. 6). However, none of these gene sets were enriched for NSCLC- and SCLC-specific known driver genes, either from IntOGen/COSMIC nor from Cell Model Passports. For brain networks, we found 113, 116, and 178 differentially strong nodes between glioblastoma and medulloblastoma samples in SSN, LIONESS and SSPGI networks respectively (Supplementary Fig. 8). Again, there was no significant enrichment for known subtype-specific drivers. There was a strong tendency towards negative LFCs for both lung and brain analyses in SSN and LIONESS networks, a phenomenon not observed during DE analysis. This observed preference is likely caused by an unbalanced group size of tumor subtype-specific samples used to construct the aggregate network, i.e., 73 NSCLC versus 12 SCLC samples and 36 glioblastoma versus 9 medulloblastoma samples, resulting in aggregate networks which are more representative of NSCLC and glioblastoma samples respectively. In SSN, LIONESS and iENA, we noticed a tendency for lower node strengths for the bigger subtype group in both lung and brain samples, while this potential bias was absent in SWEET, CSN and SSPGI (Supplementary Figs. 10–19). SWEET aims to minimize subtype group size bias through incorporation of a weighing factor reflecting genome-wide correlations across samples, resulting in similar edge weight distributions across sample groups (Supplementary Fig. 11, 17). However, some bias seemed to remain present, since also in SWEET networks, there was a slight tendency towards negative LFCs (Fig. 6c and Supplementary Fig. 8c).

### Relating single-sample networks to sample-specific molecular features

**Fig. 7:**
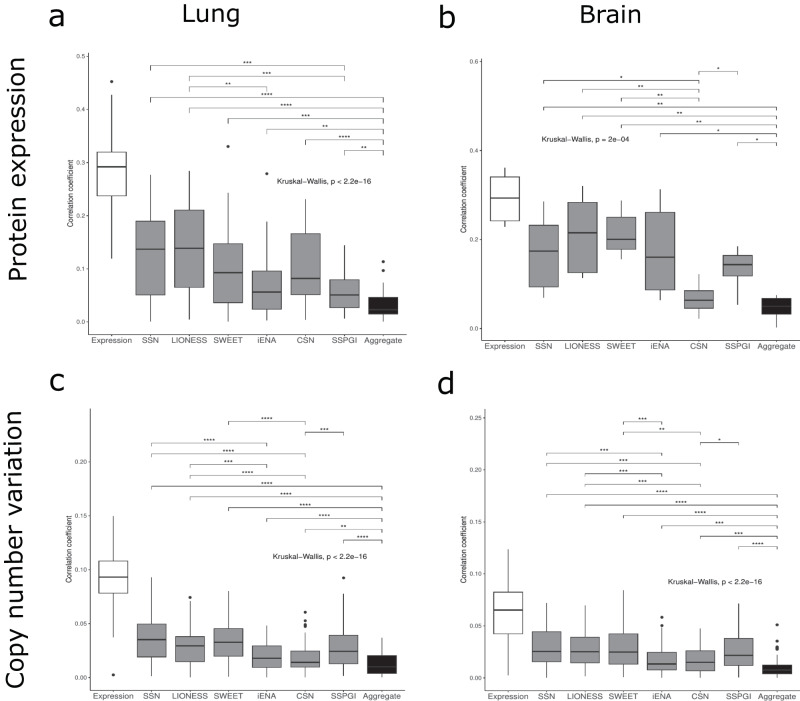
Feature-wise correlation between node strength in single-sample networks and other sample-specific omics data. **a** Lung proteomics data; **b** Brain proteomics data; **c** Lung Copy Number Variation (CNV) data; **d** Brain Copy Number Variation (CNV) data. Significant differences of the average correlation coefficients between the single-sample methods and the aggregate HumanNet network (not for expression) are shown above the boxplots (*p ≤ 0.05, **p ≤ 0.01, ***p ≤ 0.001 and ****p ≤ 0.0001, Kruskal-Wallis and post hoc Dunn test).

Finally, we assessed the biological relevance of single-sample functional gene networks by comparing them to additional CCLE omics measured on the same samples. Ideally, these single-sample networks constructed from transcriptional gene expression profiles have a higher resemblance to other sample-specific omics than the aggregate network has. We downloaded proteomics and copy number variation (CNV) data from CCLE (Methods) and assessed the correlation between node strength i.e. the sum of absolute edge weights of a node and protein abundance, and node strength and CNV (Fig. 7a–d). For the aggregate networks, we assessed correlations between node strength in the aggregate HumanNet network and proteomics/CNV measurements in each individual sample. On average, node strength in the aggregate network did not correlate well with protein abundance or CNV data, displaying correlation coefficients <0.1 for proteomics data and <0.05 for CNV data. On the other hand, for all methods, single-sample networks significantly outperformed the aggregate network for correlation of node strength to both proteomics and CNV data. Only for brain single-sample networks constructed by CSN we detected no significant difference with the aggregate network in the average correlation coefficient between node strength and protein abundance. Overall, single-sample networks from SSN, LIONESS and SWEET resulted in the largest average correlation coefficients, for both lung and brain samples, and for proteomics and copy number variation data. Together, these findings suggest that single-sample network inference methods were better in capturing sample-specific molecular features than aggregate networks and that SSN, LIONESS and SWEET single-sample networks correlated similarly and higher with sample-specific omics than the other methods, and the aggregate network.

## Discussion

The fight against highly complex and heterogeneous diseases such as cancer necessitates an in-depth understanding of disease pathobiology, at population level, but especially at the level of individual patients. Investigation of biological networks and their rewiring in disease can therefore greatly benefit the development of individualized therapeutic strategies. Although single cell technologies offer the ability of constructing networks for individual patients, there are still limitations associated with this approach, especially in the clinical setting. Bulk molecular profiling techniques on the other hand are well established and cheaper, there is a plethora of data already available, and bulk network inference algorithms have been extensively benchmarked^12^. However, bulk network inference methods construct population-level networks, representing interactions shared by most patients. Single-sample network inference methods have thus been developed to prioritize biologically meaningful information of a single individual from bulk omics data, bridging the gap towards personalized medicine, a major goal in present-day cancer research^10^.

In this study, we compared six single-sample network inference algorithms, LIONESS, SSN, SWEET, iENA, CSN and SSPGI in their construction of single-sample functional gene networks from tumor cell line transcriptomics data in the absence of normal samples. Specifically for these single-sample networks, we investigated graph properties, sample-specificity and cancer driver properties of hubs, the ability to distinguish samples of different cancer subtypes from each other, as well as their concordance with other sample-specific omics data. Although each method functions as intended in its original publication and research context, these studies understandably lack neutrality, and so far, only limited benchmarking has been performed^22,28–30^. First, we discovered that each method had different characteristics and requirements concerning in- and output data structures. The SSPGI algorithm was not scalable above 7800 genes. Also, as CSN returns binary networks comprising either zero or one as edge weights, the average number of edges within each single-sample network was lower compared to other methods. Therefore, we first pruned networks by selecting for edges present in the HumanNet reference network, and then selected the top 25 000 edges within each single-sample network to compare networks of similar size. Furthermore, edge weights ranged between highly different outer bounds in networks from very low in SSN to very high in SSPGI, so after exploring edge weight distributions we scaled them to values between [-1,1] for all methods.

Existing benchmarks focused on a limited number of features per sample, on the performance of further downstream structural control (SSC) methods, or only evaluated a limited number of network inference tools^28–30^. A comparison between LIONESS and SSN revealed that when both methods depend on the exact same aggregate network, there is an almost perfect linear relationship between edge weights of LIONESS and SSN networks for a given sample^30^. Both methods heavily rely on PCC for network construction, and construct highly similar networks. Indeed, we found that SSN and LIONESS networks had similar network topological characteristics, hub gene sets and correlation to sample-specific omics. However, it must be noted that the mathematical framework employed by LIONESS allows to make use of more advanced network inference tools than PCC^22^.

Hub genes in SSN networks, identified based on node degree, have been shown to be strongly related to cancer driver mutations^19^. However, there is no consensus regarding the number of nodes to select as hubs. While the SSN study suggests to use the top 5, 10 or 20 most connected nodes, we selected the top 200 most connected nodes and found hub gene sets to be significantly enriched for known subtype-specific driver genes. Within methods, there was a variable number of overlapping hubs between different single-sample networks and the aggregate network, with hub genes identified in CSN and SWEET networks displaying the lowest diversity across samples. As a result, hubs from these networks also had the lowest cancer subtype specificity, as most hubs regularly recurred across both subtypes. Since all single-sample networks were undirected coexpression networks, we calculated node strength as the sum of absolute edge weights. We identified most differentially strong nodes in single-sample networks of SSN, LIONESS and SSPGI, although these were not enriched for known subtype-specific cancer driver genes.

One critical remark is that the original applications of LIONESS, SSN, iENA and SSPGI used a group of healthy samples to create the aggregate network or build the edge perturbation matrix, which was not the case in this study. Paired healthy and disease samples are not always available in a clinical setting and not for all tumor types, thus we aimed to investigate the performance of these methods in the absence of control reference samples. When the aggregate network is constructed from a healthy or homogenous group of samples, each sample of interest will be compared to this aggregate network representing a healthy state. One can thus argue that the construction of an aggregate network from a heterogenous group of samples will eventually result in less explicit differences between the aggregate and the single-sample networks. Due to the unbalanced tumor subtype sample numbers during the construction of aggregate lung (73 NSCLC versus 12 SCLC samples) and brain (36 glioblastoma versus 9 medulloblastoma samples) networks, the final aggregate networks were dominated by the larger sample group. As a result, we observed a tendency for higher average node strengths for samples belonging to the underrepresented sample group for SSN, LIONESS and iENA. Also, we noticed a strong tendency towards negative LFCs in comparisons of the node strengths between subtype sample groups for LIONESS and SSN networks. Also the higher proportions of subtype-specific hubs in SSN, LIONESS and iENA networks could potentially be attributed to this potential bias. In the recent study of the single-sample network inference method SWEET, the authors also noticed that sample size differences between intrinsic subpopulations may cause a network size bias in the statistical perturbation model for the SSN method, in the statistical dependency model for the CSN method and in the model of removing a single sample from an aggregate network for the LIONESS method^27^. Adversely, SWEET includes a weighting factor during edge weight calculation that reflects genome-wide sample-to-sample correlations. However, in our study this resulted in highly similar single-sample networks, as we observed high similarity of hub gene sets, low hub subtype-specificity, and only a single differentially strong node in NSCLC vs SCLC or medulloblastoma vs glioblastoma single-sample networks. Nevertheless, SWEET, together with SSN and LIONESS, was one of the methods where node strengths correlated the best with single-sample proteomics and CNV data. It must be noted that whereas SWEET employs a Z-test on the fully connected network to select edges and build the final single-sample networks, we opted to use SWEET with a selection of the top 25 000 edges, as we did for the other methods. SWEET will weigh edges of subtype samples that are overrepresented more, because the difference of these edge weights to the edge weights of the aggregate network becomes less. Upon selecting the edges with the top 25 000 highest weights, SWEET single-sample networks together will therefore consist of more balanced edge weights reflecting all subtypes as opposed to LIONESS that will favor the edge weights of underrepresented subtypes.

Hence, we advocate for the careful assembly of aggregate networks with subgroups of similar size, especially when using SSN, LIONESS or iENA. Ideally, also covariates are taken into account, although this is not possible in the correlation framework employed by SSN, LIONESS, SWEET or iENA, or the frameworks employed by CSN and SSPGI^39^. The recent single-sample network inference method DysRegNet, which is based on an aggregate network of normal control samples, employs linear models using TF expression as an explanatory variable for target gene expression, which allows to also incorporate known covariates such as sex, age, or origin of the sample^39^.

Overall, there is a lack of ground truth data which makes a true benchmark study difficult. Instead, we explored the relationship between single-sample networks and other omics data modalities, namely proteomics and CNV, at the sample level. We found that single-sample networks inferred by all methods outperformed the aggregate network regarding correlation to sample-specific omics. Furthermore, we demonstrated this correlation difference with two independent omics data, proteomics and CNV, reinforcing that single-sample networks provide sample-specific information that is not present in the aggregate network, hence their added value. SSN, LIONESS and SWEET showed a higher correlation to sample-specific omics than the other methods. Correlation to gene expression data was even higher, however, gene expression data in itself cannot provide the additional level of systems biological insights as provided by network analysis through e.g. hub gene analysis or differential node strength. Moreover, clusters of nodes in coexpression networks often represent biological entities that function together in the same process^9^. Also, we heavily relied on cancer subtype annotations of CCLE cell lines during our hub gene and differential strength analyses. Yet, clustering of samples based on expression data showed that these annotations might not be ideal, as several samples clustered together with other subtypes. These issues have previously been addressed^33^, and we used a specific approach to include the most relevant cell lines in our study.

**Fig. 8:**
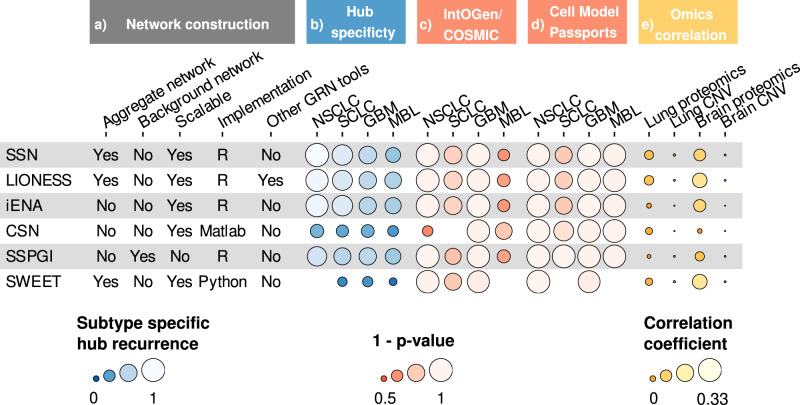
Summary of single-sample networks evaluation. Overview of the six single-sample network inference tools compared in this study. The single-sample networks were evaluated in this study for their methodology and implementation in **a** Network construction, their subtype-specific hub recurrence in **b** Hub specificity, their enrichment for known cancer driver genes in both IntOGen/COSMIC and Cell Model Passports in **c** IntOGen/COSMIC and **d** Cell Model Passports, and their correlation to other sample-specific omics data in **e** Omics correlation. ‘Aggregate network’ and ‘background network’ reflect whether these are required by each tool. ‘Scalable’ reflects whether a tool could be run on the complete set of highly variable genes. ‘Other GRN tools’ reflects whether a tool can be used in combination with advanced regulatory network inference tools other than PCC. ‘Edge weight bias’ reflects whether we observed an edge weight bias due to unbalanced sample groups while constructing the aggregate network. Subtype-specific hub recurrence was calculated by dividing the average recurrence of subtype-specific hubs by the average recurrence of non-subtype-specific hubs, scaled between 0 and 1. Enrichment columns reflect 1 – *p* values for enrichment of different hub gene sets as described in Fig. 5 and Supplementary Fig. 7 (hypergeometric test). Omics correlation reflects the correlation to other omics as described in Fig. 7.

In conclusion, we have constructed single-sample networks for 86 lung and 69 brain cancer cell lines from CCLE, using six different single-sample network inference methods. Several network pruning steps were required to make networks comparable. For all methods, we found that hub genes were enriched for known cancer subtype-specific driver genes and node strengths of single-sample networks correlated better to sample-specific omics than the traditional bulk aggregate network, suggesting that single-sample networks are a valuable tool for personalized medicine (Fig. 8). Overall, CSN and SWEET performed worse than other methods in hub analyses such as hub specificity and enrichment of known drivers. SWEET single-sample networks seemed to be highly similar to each other regarding hub gene sets and a lack of differentially strong nodes, which suggest the inclusion of a weighting factor during single-sample network inference removes a significant portion of variability. Also CSN networks might suffer from high similarity across samples due to the binary nature of edge weights. Based on correlation of the node strengths of single-sample networks to sample-specific proteomics and CNV data, SSN, LIONESS and SWEET performed best in providing sample-specific information (Fig. 8). For SSN, LIONESS and iENA, it is important to balance different sample groups within the samples under study, since these methods seemed to have a bias for sample group size.

Hence, from our study, we conclude that most of the single-sample network inference methods are able to reflect sample-specific biology better than aggregate networks for use-cases were ‘normal tissue’ samples are absent. However, single-sample network inference remains a very challenging task since information gain depends on only one datapoint per gene, and we showed that algorithmic choices can have strong influences on the outcome. While these methods represent a valuable resource for personalized medicine and precision oncology, we recommend that any generated hypothesis should be carefully interpreted and experimentally validated.

## Methods

### Data and cell line selection

Expression read counts and metadata were downloaded from the DepMap (Cancer Dependency Map) website (20Q4 version 2 release)^31,32,40^. Expression data were available for 84 primary brain cancer and 189 primary lung cancer cell lines. For both tumor types, outlier cell lines were excluded in two ways. First, only cell lines with Spearman correlation of expression profiles greater than 0.55 to real tumor tissue, as outlined in a pan-cancer comparison of CCLE cell lines and TCGA tumor samples^33^, were kept to ensure biological interpretability. Second, cell lines strongly differing from the other cell lines of the same tumor type were removed by clustering to exclude unrepresentative samples for a given tissue. Clustering was done with the hclust function using average agglomeration, with tree cutting at 95% of the maximum height of the tree in R (version 3.6). Clusters with less than 3 samples were removed. After filtering, 67 brain and 86 lung cancer cell lines were retained for single-sample network inference.

### Expression data preprocessing

The RNA-seq count data was processed using EdgeR^41^ in R (version 3.6). Raw counts were filtered to keep only genes with counts per million (cpm) greater than 1 in at least one sample. Next, raw counts were normalized by library size, converted into cpm and log-transformed. We selected 7942 and 9252 highly variable genes (HVG; variance >2.75 over all samples per tumor type), respectively, for brain and lung as input for single-sample network inference (HVG selection). Due to lack of scalability, only 7800 highly variable genes were retained for SSPGI for both tumor types (see SSPGI method section). Finally, scaling and centering were performed per gene. Heatmaps were constructed after calculating Spearman correlations between samples and applying Ward linkage using ComplexHeatmap^42^.

### Aggregate networks and network visualization

Aggregate networks were constructed separately for brain and lung samples using PCC after HVG selection. These fully connected coexpression networks were subjected to pruning for edges in the HumanNet background network, an integrated functional gene network^34^. We choose to work with the HumanNet-XN v2 network (https://www.inetbio.org/humannetv2/), which contains 17 929 genes and 525 537 edges representing physical protein-protein interactions, functional associations substantiated by different omics data and interologs from other species and co-citation links. We then selected the top 25 000 edges based on edge weights in these networks.

### Cancer driver genes

Lists of known cancer drivers i.e. genes which contain mutations that have been causally implicated in cancer, for different lung and brain cancer subtypes were downloaded from the IntOGen website (19/06/2023, https://www.intogen.org/search) and the COSMIC/Cancer Gene Census website (19/06/2023, https://cancer.sanger.ac.uk/cosmic/census). Also, for each CCLE cell line included in this study, specific known cancer drivers were downloaded from Cell Model Passports (19/10/23, https://cellmodelpassports.sanger.ac.uk/). These cancer drivers were then grouped per sample group and considered subtype-specific drivers.

### Single-sample network inference methods

Table 2 provides an overview of the different single-sample network inference methods used in this study. We slightly modified several methods to run them with PCC as underlying network inference method, without ‘normal tissue’ reference samples, as well as with different or without background networks.

**Table 2:** Overview of the different single-sample network inference methods used in this study

Method	Underlying concept	Remarks
SSN^19^	Differential correlation between reference network and reference network + sample of interest $$\begin{array}{l}\Delta {PCC}={{PCC}}_{r+1}-{{PCC}}_{r}\\{with\; PCC}({x}_{i},{x}_{j})=\frac{{Covariance}({x}_{i},{x}_{j})}{\sqrt{{Variance}\left({x}_{i}\right){Variance}({x}_{j})}}\end{array}$$	All cancer samples of specific tumor type as reference, no normal samples No background network such as STRING, no significance testing of the edges
LIONESS^22^	Linear interpolation ${e}_{i,j}^{q}=N\left({e}_{i,j}^{\alpha }-{e}_{i,j}^{\alpha -q}\right)+{e}_{i,j}^{\alpha -q}$ With e edge weight in respectively all samples network (α) or all samples network without sample of interest q (α - q) and N scaling factor inversely proportional to the total number of samples	PCC as aggregate network inference method
IENA^24^	Single-sample correlation ${sPCC}\left({x}_{i},{x}_{j}\right)=\frac{{{Covariance}}^{r}\left({x}_{i},{x}_{j}\right)}{\sqrt{{{Variance}}^{r}\left({x}_{i}\right){{Variance}}^{r}\left({x}_{j}\right)}}$ ${with}\,{{Covariance}}^{r}({x}_{i},{x}_{j})=({x}_{i}-{\mu }_{i}^{r})({x}_{j}-{\mu }_{j}^{r})$ ${with}\,{{Variance}}^{r}\left({x}_{i}\right)=\frac{1}{N}\mathop{\sum }\nolimits_{i=1}^{N}{({x}_{i}-{\mu }_{i}^{r})}^{2}$ With mean and variance for genes x_i_ and x_j_ taken from the reference ^r^	Only sPCC node-networks from iENA, not shPCC edge-networks All cancer samples of specific tumor type as reference, no normal samples
CSN^26^	Local gene-gene association based on statistical independency model ${\rho }_{{ij}}^{s}=\frac{{n}_{{ij}}^{s}}{n}-\frac{{n}_{i}^{s}}{n}.\frac{{n}_{j}^{s}}{n}$ With ρ statistic of edge i-j in cell / sample s, n is the number of cells / samples with expression of gene i, j or both as plotted in scatter diagrams, and n_i_^s^ = n_j_^s^ = 0.1n	Binary output Developed for single cell Scatter diagrams per gene pair considering all cancer samples
SSPGI^25^	Edge perturbation based on gene expression rank subtraction $$\begin{array}{l}{\Delta }_{e,s}={\delta }_{e,s}-\bar{{\delta }_{e}}\\{\delta }_{e,s}={r}_{i,s}-{r}_{j,s}\end{array}$$ With expression rank r, delta rank matrix $\delta$ and benchmark delta rank vector $\bar{\delta }$ for an edge e between genes i and j within a sample s	HumanNet as background network, not Reactome Not scalable, up to 7800 genes Benchmark delta rank vector calculated using all cancer samples of specific tumor type as reference, no normal samples
SWEET^10^	Linear interpolation with genome-wide sample weight W^q^ ${W}^{q}=\frac{{\mu }_{{PCC}}^{q}-\min {\mu }_{{PCC}}+x}{\max {\mu }_{{PCC}}-\min {\mu }_{{PCC}}+x}$ With ${\mu }_{{PCC}}^{q}$ the mean of PCCs between sample q and all other n samples, ${\mu }_{{PCC}}$ is the set of average PCCs for n samples and x is a constant value of 0.01 ${e}_{i,j}^{q}={W}^{q}\times n\times K({e}_{i,j}^{\alpha +q}-{e}_{i,j}^{\alpha })+{e}_{i,j}^{\alpha }$ With e edge weight in respectively all samples network (α) or all samples network plus sample of interest q (α+q) and K a balance parameter of 10%	No significance testing of the edges

We slightly modified several methods because we ran them with PCC as underlying network inference method, without ‘normal tissue’ reference samples, and with different or without background networks. This allowed making a consistent comparison between methods.

In **SSN** (Single-Sample Network), a reference PCC network is first generated based on transcriptome data of several reference samples, usually normal tissue samples. Here we used all selected cell lines for a specific tumor type, with the sample of interest each time omitted as a reference. Then, the same is done for all the reference samples plus the sample of interest to generate the so-called perturbed network. Finally, these two networks are subtracted from each other. The significance of p-values is not considered as we prune all the networks in the same way using HumanNet and 25k HumanNet (Fig. 2)^19^. Although there is a SSN Python implementation online, we made our own R implementation for ease of use with the above-mentioned modifications.

In **LIONESS**, linear interpolation is performed on the edge weights of two networks, here constructed by PCC, the first containing all samples, the second containing all samples except for the sample of interest^22^. We used the LIONESS function from the LIONESS R package (https://github.com/mararie/LIONESSR). This function creates one edge list file for all samples in the input expression dataset. We used the single-sample PCC calculation in the iENA node-network^24^, where the PCC between two genes in a single-sample is calculated using mean and variance in a reference group, usually normal tissue samples, but here all selected cell lines for a specific tumor type. A customized implementation of the algorithm in R was made because no source code was provided with the original publication.

The **Cell-Specific Network** (CSN) was developed originally to infer gene association networks for single cells. Still, it can also be applied to bulk data to infer single-sample networks^26^. It generates a binary output, where gene-gene interactions are considered present (1) or not present (0). CSN is based on statistical dependency. For each gene pair, an expression scatter diagram is made in which each dot represents one cell or sample. Next, within each plot, the neighborhood of each sample is identified using a predefined rectangular distance threshold along both axes. The number of neighboring cells or samples in these neighborhoods (n_x_, n_y_ and n_xy_) divided by the total number of cells or samples n are estimates of the marginal density function of x and y and the joint density function of x and y, respectively. These are used to define a statistic from -1 to 1, which follows a normal distribution given that gene x and gene y are independent. Because the mean and the standard deviation of this normal distribution are known, this statistic can be used to calculate a p-value that, in case of significance, rejects the null hypothesis that gene x and y are independent in sample k and form an edge. The MATLAB code for this method is provided on the papers GitHub page (https://github.com/wys8c764/CSN).

**SSPGI** calculates edge perturbation values^25^. First, the gene expression matrix is converted into a rank matrix by ranking the genes according to the expression value in each sample. Second, a delta rank matrix is calculated by subtracting the ranks of any two genes connected by a given edge in the background network. The original publication created a background network based on all gene interactions in the Reactome pathway database^43^. In theory, the required background network could contain all possible edges between all genes in all samples, but in practice this is not feasible due to the lack of scalability of SSPGI. We could run SSPGI only on 7800 genes and with HumanNet given as background network, which caused no other interactions being calculated than the ones present int HumanNet. Including more genes, or all possible edges as background caused the method to terminate with an error. For all methods we worked on a high performance computing infrastructure on an Dual Intel Xeon Gold 6420 CPU cluster using one node with a usable memory of 700 GBM and 2×18 cores. As within-sample delta ranks of gene pairs are stable under normal conditions, a benchmark delta rank vector is calculated using the mean rank of all genes across the reference group of normal samples. However, we built this benchmark delta rank vector using all selected cell lines for a specific tumor type. Finally, the edge perturbation matrix is created by subtracting every sample’s benchmark delta rank vector from the delta rank matrix. The authors provide the SSPGI implementation written in R on their GitHub page (https://github.com/Marscolono/SSPGI).

**SWEET** constructs a single-sample network for each sample S_p_ based on the gene expression of n case samples. First, SWEET calculates a genome-wide sample-to-sample correlation matrix. The average PCC for each sample S is then used to calculate a genome-wide sample weight W_S_. Second, an aggregate network is constructed using PCCs as edge weights. A perturbed network is then inferred by creating a copy of the expression profile of sample S, and calculating PCCs between all genes for n + 1 samples. Finally, the difference between the aggregate and perturbed network is calculated and integrated with genome wide sample weights W to construct n single-sample networks. The significance level of each edge is later evaluated using a z-test, and all edges with a score larger than the significance level are removed. However, in our implementation we omitted this last step, and selected the top 25 000 edges in each single-sample network instead, so that networks constructed by different methods were comparable in size. Due to a more recent genome annotation used to convert Entrez IDs in the HumanNet network to gene symbols, SWEET networks were slightly larger than SSN, LIONESS, iENA, CSN and SSPGI before selection of the top 25 000 edges.

Outputs from each single-sample network inference method were converted into a dataframe with edges as rows and samples as columns, generating a uniform format as provided by the LIONESS algorithm. In a subsequent step, we filtered the edges of the other single-sample networks obtained by SSN, LIONESS, SWEET, iENA and CSN based on the HumanNet background network, as explained for the aggregate network. Finally, we selected the top 25 000 edges in each single-sample network constructed by SNN, LIONESS, SWEET, iENA and SSPGI, in order to make them comparable in size to the average CSN network.

### Analysis of network topology

The average edge weight distribution was plotted for each method using ggplot2’s *geom_density* function in R^44^. The average weight of an edge was determined by calculating the average weight of a given edge across all samples, ignoring entries for which the given edge was not present in the single-sample network. For plotting the weight density distribution of all edges, the weights of all non-zero edges were concatenated into a single weight vector. We used the igraph package in R to determine network characteristics^45^. Clustering coefficients were calculated using the *transitivity* function with type *average*, network densities using the *edge_density* function without considering loops. Node betweenness was calculated using the *estimate_betweenness* function while treating the network as an undirected graph. As this is a node-specific characteristic, we calculated the mean value across all nodes within each sample. Edge betweenness was determined using the *betweenness* function with the same parameters as for node betweenness. Finally, the *diameter* and *count_components* functions were used to calculate network diameter and the number of connected components, respectively.

### Principal component analysis

The node strength was calculated as the sum of absolute edge weights for each node^37^. Node strength matrices were transposed so rows represented samples and columns represented nodes, after which R’s *prcomp* function was applied. Plots were drawn using the *autoplot* function in ggplot2, and dots were colored according to the sample cancer subtype.

### Hub gene analysis

Hub genes were identified as the top 200 most connected nodes in each top 25k single-sample network. Enrichment for known cancer driver genes was assessed under a hypergeometric distribution using all genes present in the network as background. Violin plots were made visualizing the recurrence of hubs in all samples as well as all samples within a disease subtype. Regularly recurring hubs were defined as hubs recurring in at least 75% of networks in a sample group per method.

### Differential node strength

Since LIONESS, SSN, iENA, CSN, SSPGI and SWEET produce single-sample networks with different ranges of edge weights, we performed a within-sample normalization to scale edge weights within [-1, 1]. The differential node strength between sample subgroups was evaluated by calculating the sum of absolute edge weights for each node and applying linear modeling with an Empirical Bayes procedure, as implemented in the limma package^46^. The differential strong nodes were identified having an absolute log-fold change (LFC) > 1 and an adjusted p-value < 0.05 (Benjamini & Hochberg correction). Enrichment for known cancer driver genes was assessed by a hypergeometric distribution using a combined list of all known driver genes per tissue and all genes present in the network as background.

### Comparison to other omics data

Normalized proteomics datasets were downloaded from the CCLE website (protein_quant_current_normalized.csv, version 20Q4), and cell lines were matched to samples present in single-sample networks for lung and brain, separately. Rows with duplicate gene symbols were removed. As these data were already normalized, no further preprocessing was applied. Next, we selected samples with matching proteomics data from the preprocessed expression dataset. The node strength i.e. the sum of absolute edge weights per node was calculated in all single-sample networks and the aggregate networks. We then calculated PCCs between proteomics data and the node strength of all nodes in the single-sample networks for each matching sample, and between the node strengths of the aggregate network and each individual proteomics sample. Copy number variation data was also downloaded from the CCLE website (20Q4_v2_CCLE_gene_cn.csv). We applied the same procedure to copy number variation data. Results were plotted using ggplot2^44^.

### Funkyheatmap

The funky heatmap was plotted using funkyheatmap package in R^47^.

### Reporting summary

Further information on research design is available in the Nature Research Reporting Summary linked to this article.

### Supplementary information

Supplemental material

Reporting summary

## Data Availability

No new data was generated for this study.

## Code availability

All scripts used to construct and analyze the aggregate and single-sample functional gene networks in this study can be found at https://github.com/CBIGR/single_sample_networks.

## References

[1] Singer J (2019). Bioinformatics for precision oncology. Brief. Bioinform..

[2] Erbe R, Gore J, Gemmill K, Gaykalova DA, Fertig EJ (2022). The use of machine learning to discover regulatory networks controlling biological systems. Mol. Cell.

[3] Ozturk K, Dow M, Carlin DE, Bejar R, Carter H (2018). The emerging potential for network analysis to inform precision. Cancer Med. J. Mol. Biol..

[4] The DREAM5 Consortium. (2012). Wisdom of crowds for robust gene network inference. Nat. Methods.

[5] Mercatelli D, Scalambra L, Triboli L, Ray F, Giorgi FM (2020). Gene regulatory network inference resources: a practical overview. *Biochim. Biophys*. Acta BBA-Gene Regul. Mech..

[6] Delgado FM, Gómez-Vela F (2019). Computational methods for Gene Regulatory Networks reconstruction and analysis: A review. Artif. Intell. Med..

[7] Vermeirssen V (2009). Transcription regulatory networks in Caenorhabditis elegans inferred through reverse-engineering of gene expression profiles constitute biological hypotheses for metazoan development. Mol. Biosyst..

[8] Vermeirssen V, De Clercq I, Van Parys T, Van Breusegem F, Van de Peer Y (2014). Arabidopsis ensemble reverse-engineered gene regulatory network discloses interconnected transcription factors in oxidative stress. Plant Cell.

[9] Loers JU, Vermeirssen V (2022). SUBATOMIC: a SUbgraph BAsed mulTi-OMIcs clustering framework to analyze integrated multi-edge networks. BMC Bioinforma..

[10] van der Wijst MGP, de Vries DH, Brugge H, Westra H-J, Franke L (2018). An integrative approach for building personalized gene regulatory networks for precision medicine. Genome Med..

[11] Yurkovich JT, Tian Q, Price ND, Hood L (2020). A systems approach to clinical oncology uses deep phenotyping to deliver personalized care. Nat. Rev. Clin. Oncol..

[12] Nguyen H, Tran D, Tran B, Pehlivan B, Nguyen T (2020). A comprehensive survey of regulatory network inference methods using single cell RNA sequencing data. Brief. Bioinform..

[13] Vitali F (2017). Developing a ‘personalome’ for precision medicine: emerging methods that compute interpretable effect sizes from single-subject transcriptomes. Brief. Bioinform..

[14] Gardeux V (2014). ‘N-of-1- *pathways* ’ unveils personal deregulated mechanisms from a single pair of RNA-Seq samples: towards precision medicine. J. Am. Med. Inform. Assoc..

[15] Wang H (2015). Individual-level analysis of differential expression of genes and pathways for personalized medicine. Bioinformatics.

[16] Xie J (2020). Identification of population-level differentially expressed genes in one-phenotype data. Bioinformatics.

[17] Alvarez MJ (2016). Functional characterization of somatic mutations in cancer using network-based inference of protein activity. Nat. Genet..

[18] Buschur KL, Chikina M, Benos PV (2020). Causal network perturbations for instance-specific analysis of single cell and disease samples. Bioinformatics.

[19] Liu X, Wang Y, Ji H, Aihara K, Chen L (2016). Personalized characterization of diseases using sample-specific networks. Nucleic Acids Res..

[20] Zhu K, Pian C, Xiang Q, Liu X, Chen Y (2020). Personalized analysis of breast cancer using sample-specific networks. PeerJ.

[21] Hu F, Wang Q, Yang Z, Zhang Z, Liu X (2020). Network-based identification of biomarkers for colon adenocarcinoma. BMC Cancer.

[22] Kuijjer ML, Tung MG, Yuan G, Quackenbush J, Glass K (2019). Estimating Sample-Specific Regulatory Networks. iScience.

[23] Lopes-Ramos CM (2018). Gene Regulatory Network Analysis Identifies Sex-Linked Differences in Colon Cancer Drug Metabolism. Cancer Res..

[24] Yu X (2017). Individual-specific edge-network analysis for disease prediction. Nucleic Acids Res..

[25] Chen Y, Gu Y, Hu Z, Sun X (2021). Sample-specific perturbation of gene interactions identifies breast cancer subtypes. Brief. Bioinform..

[26] Dai H, Li L, Zeng T, Chen L (2019). Cell-specific network constructed by single-cell RNA sequencing data. Nucleic Acids Res..

[27] Chen H-H (2023). SWEET: a single-sample network inference method for deciphering individual features in disease. Brief. Bioinform..

[28] Guo W-F (2019). A novel network control model for identifying personalized driver genes in cancer. PLOS Comput. Biol..

[29] Guo W-F (2021). Performance assessment of sample-specific network control methods for bulk and single-cell biological data analysis. PLOS Comput. Biol..

[30] Jahagirdar S, Saccenti E (2021). Evaluation of Single Sample Network Inference Methods for Metabolomics-Based Systems. Med. J. Proteome Res..

[31] Barretina J (2012). The Cancer Cell Line Encyclopedia enables predictive modeling of anticancer drug sensitivity. Nature.

[32] Ghandi M (2019). Next-generation characterization of the Cancer Cell Line Encyclopedia. Nature.

[33] Yu K (2019). Comprehensive transcriptomic analysis of cell lines as models of primary tumors across 22 tumor types. Nat. Commun..

[34] Hwang S (2019). HumanNet v2: human gene networks for disease research. Nucleic Acids Res..

[35] Kuijjer, M. L. & Glass, K. *Reconstructing Sample-Specific Networks using LIONESS*. 10.1101/2021.09.27.461954 (2021)

[36] Davis JD, Voit EO (2019). Metrics for regulated biochemical pathway systems. Bioinformatics.

[37] Wang, M., Wang, H. & Zheng, H. A Mini Review of Node Centrality Metrics in Biological Networks. *Int. J. Netw. Dyn. Intell*. 99–110 10.53941/ijndi0101009 (2022)

[38] Lopes-Ramos CM (2020). Sex Differences in Gene Expression and Regulatory Networks across 29 Human Tissues. Cell Rep..

[39] Lazareva, O. et al. DysRegNet: Patient-specific and confounder-aware dysregulated network inference. *bioRxiv* 2022–04 (2022). 10.1111/bph.17395 39631757

[40] Dempster JM (2019). Agreement between two large pan-cancer CRISPR-Cas9 gene dependency data sets. Nat. Commun..

[41] Robinson MD, McCarthy DJ, Smyth GK (2010). edgeR: a Bioconductor package for differential expression analysis of digital gene expression data. Bioinformatics.

[42] Gu Z, Eils R, Schlesner M (2016). Complex heatmaps reveal patterns and correlations in multidimensional genomic data. Bioinformatics.

[43] Gillespie M (2022). The reactome pathway knowledgebase 2022. Nucleic Acids Res..

[44] Wickham, H. ggplot2: elegant graphics for data analysis Springer-Verlag New York; 2009. *Prepr. At* (2016).

[45] Csardi G, Nepusz T (2006). The igraph software package for complex network research. Inter. J. Complex Syst..

[46] Ritchie ME (2015). limma powers differential expression analyses for RNA-sequencing and microarray studies. Nucleic Acids Res..

[47] Saelens W, Cannoodt R, Todorov H, Saeys Y (2019). A comparison of single-cell trajectory inference methods. Nat. Biotechnol..

