# Supplementary material for: Evaluation of single-sample network inference methods for precision oncology
Source: NPJ Syst Biol Appl. 2024 Feb 15;10:18. doi: 10.1038/s41540-024-00340-w (PMC10869342; doi:10.1038/s41540-024-00340-w)
Supplement: Supplementary file 1 — Supplemental material [file 41540_2024_340_MOESM1_ESM.pdf]

Supplementary information

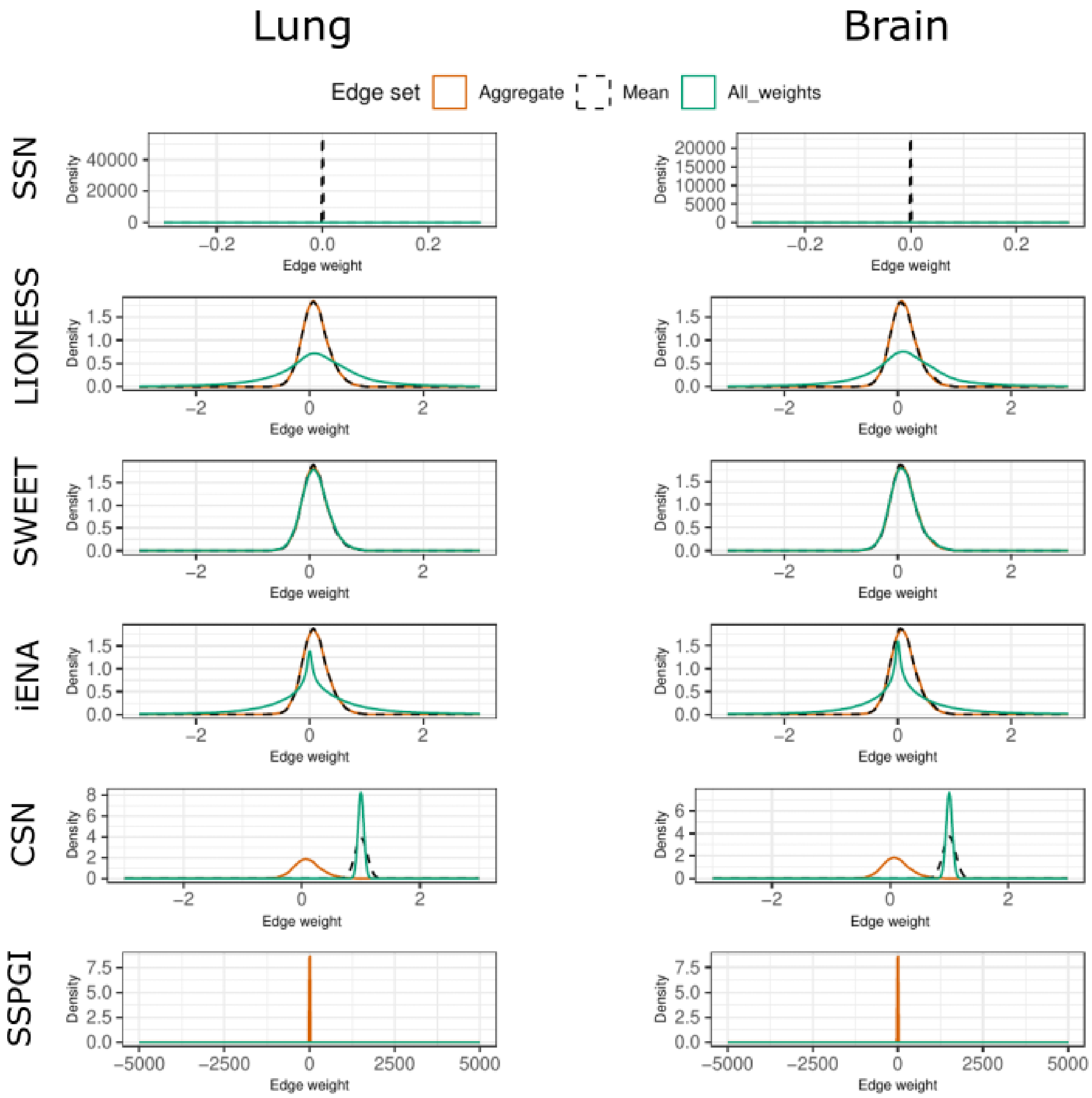

Supplementary Figure 1. Single-sample networks constructed by SSN, LIONESS, SWEET, iENA, CSN and SSPGI are characterized by distinct edge weight distributions. *Edge weight distributions were plotted after selection for edges present in the HumanNet network, for the aggregate network (orange), the mean edge weight for every single edge (only using non-zero edges) (black) and a concatenation of all weights across every sample (green).*

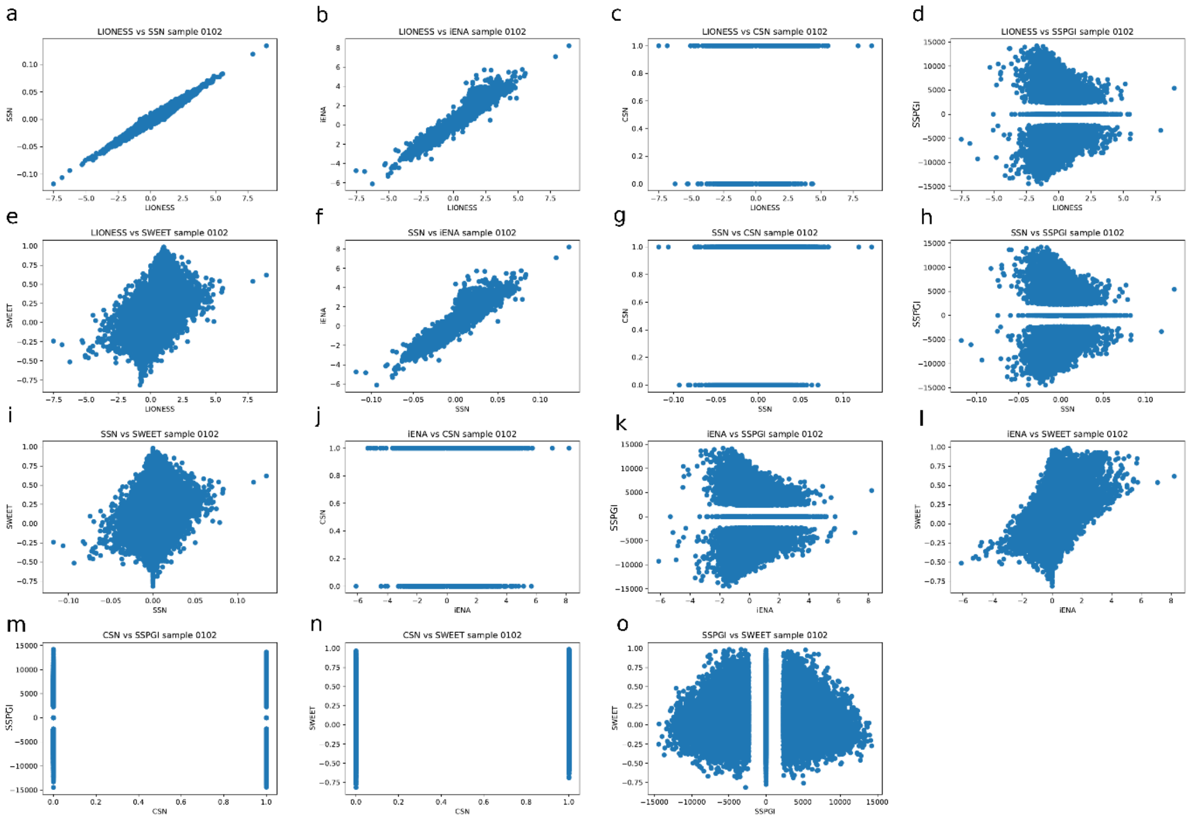

Supplementary Figure 2. Scatter plot of edge weights in single-sample networks for sample 0102. The signs of the edge weights are consistent between SSN, LIONESS, iENA and mostly SSPGI, whereas CSN has binary edge weights that are more difficult to compare to the other methods. *a) LIONESS vs SSN; b) LIONESS vs iENA; c) LIONESS vs CSN; d) LIONESS vs SSPGI; e) LIONESS vs SWEET; f) SSN vs iENA; g) SSN vs CSN; h) SSN vs SSPGI; i) SSN vs SWEET; j) iENA vs CSN; k) iENA vs SSPGI; l) iENA vs SWEET; m) CSN vs SSPGI; n) CSN vs SWEET; o) SSPGI vs SWEET. Only 1 sample is shown, results are similar for all samples.*

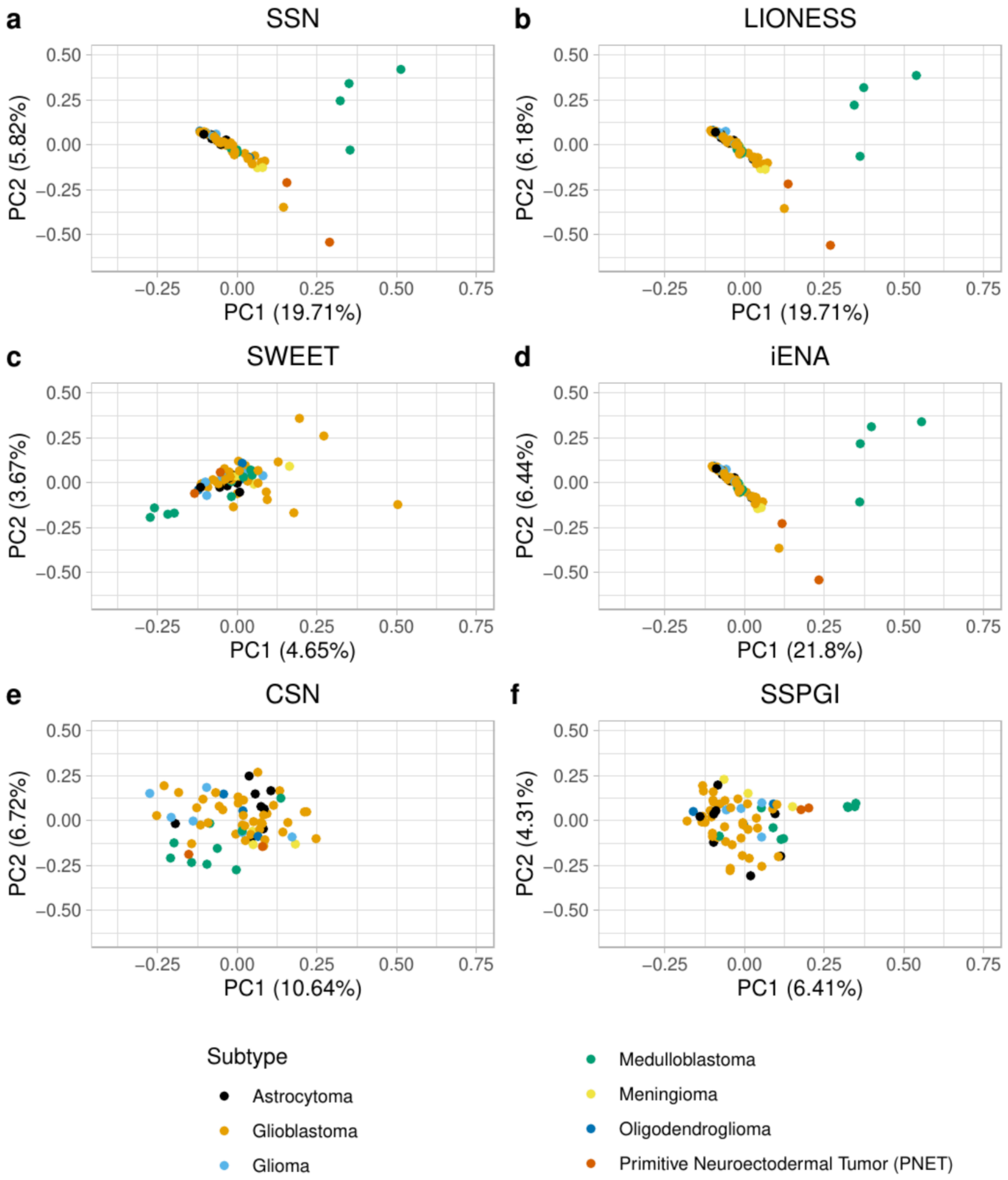

Supplementary Figure 3. Visualization of brain samples after projecting the node strengths i.e. the sum of absolute edge weights of the single-sample networks onto their first two principal components. *We performed PCA analysis on top 25k brain networks constructed using SSN (**a**), LIONESS (**b**), SWEET (**c**), iENA (**d**), CSN (**e**) and SSPGI (**f**). Each dot represents one single-sample network constructed from a cell line corresponding to a given cancer subtype. (PCA: principal component analysis)*

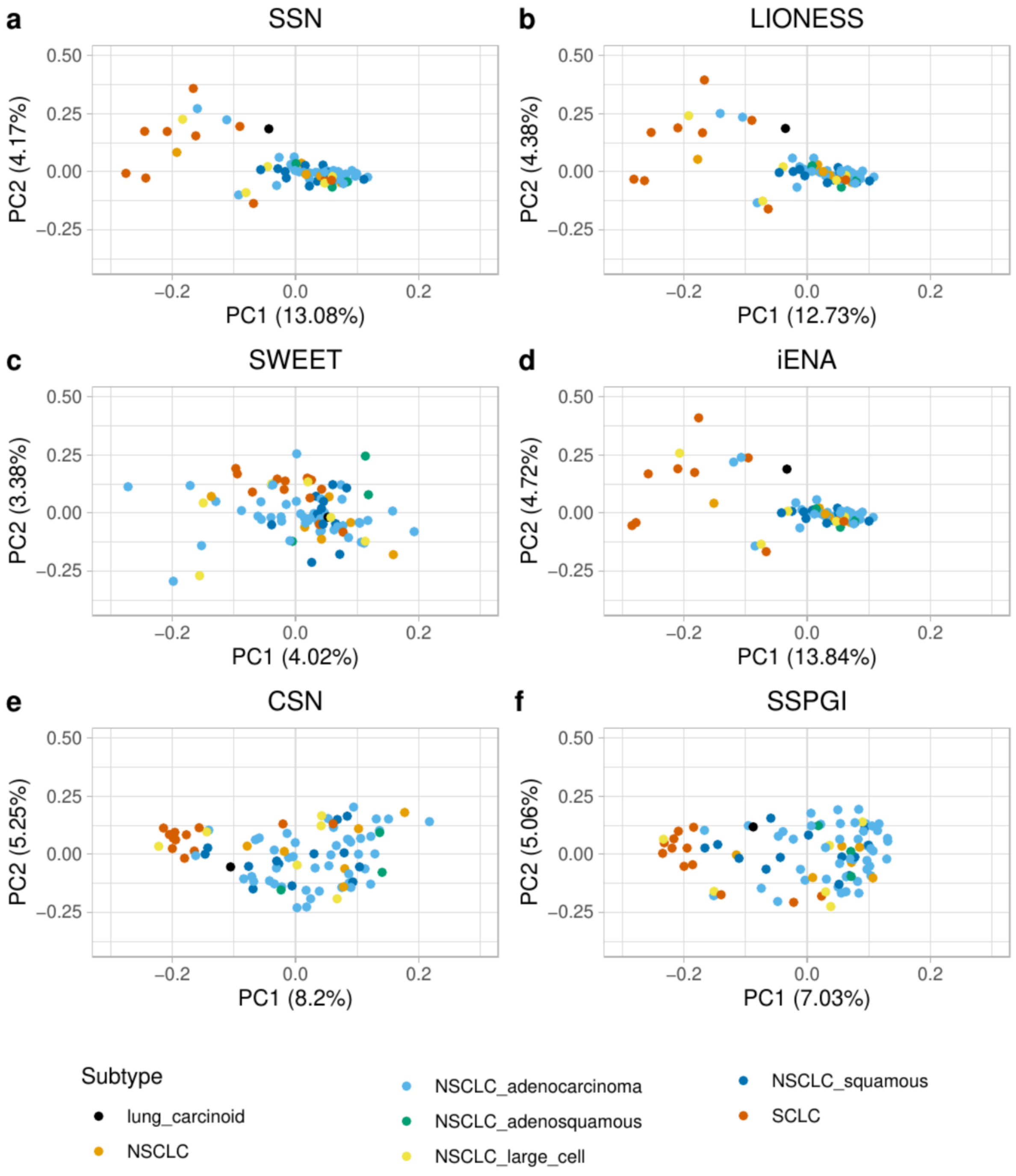

Supplementary Figure 4. Visualization of lung samples after projecting the node strengths i.e. the sum of absolute edge weights of the single-sample networks onto their first two principal components. *We performed PCA analysis on top 25k lung networks constructed using SSN (**a**), LIONESS (**b**), SWEET (**c**), iENA (**d**), CSN (**e**) and SSPGI (**f**). Each dot represents one single-sample network constructed from a cell line corresponding to a given cancer sub-subtype. (NSCLC = non-small cell lung carcinoma, SCLC = small cell lung carcinoma; PCA: principal component analysis)*

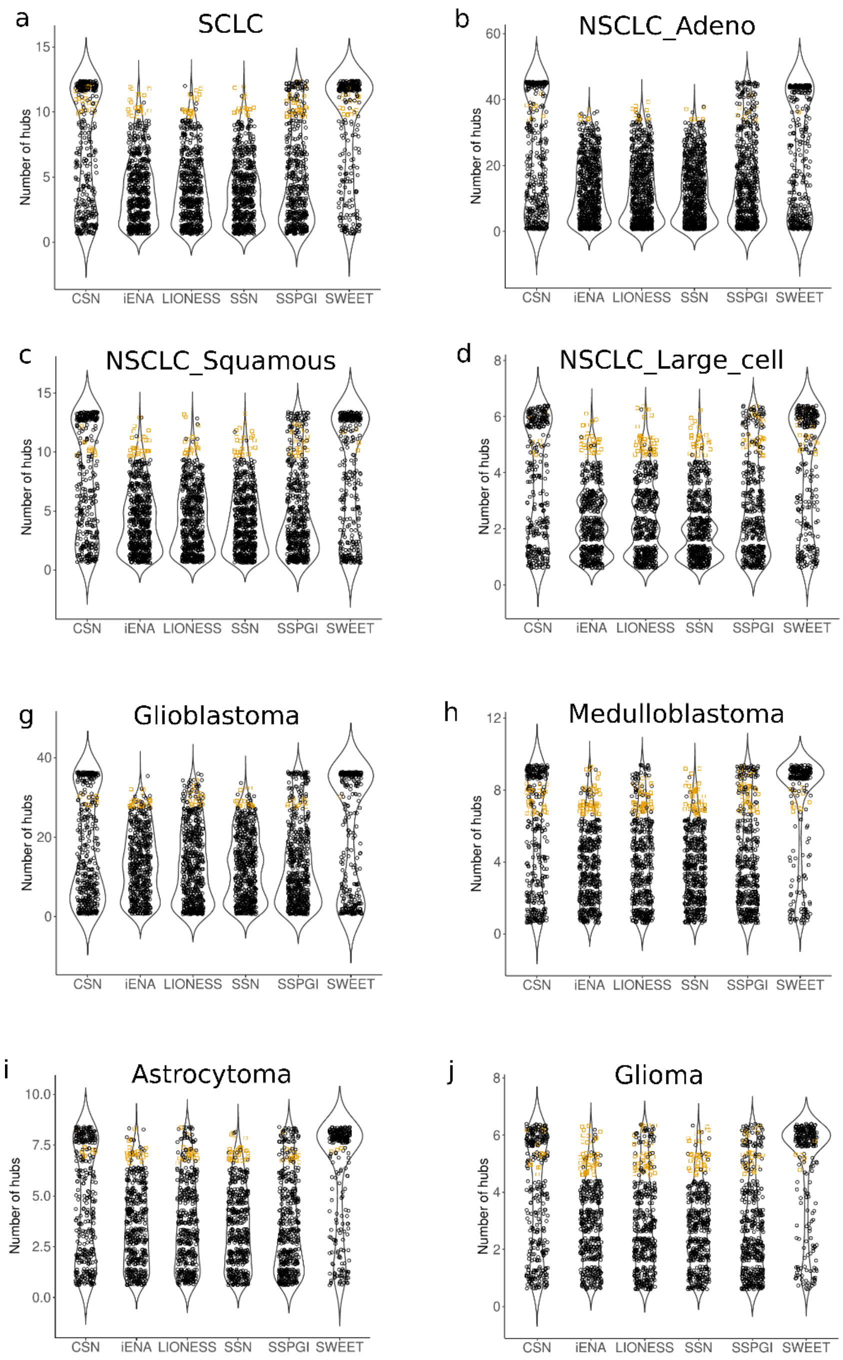

Supplementary Figure 5. Hub recurrence in single-sample networks. *Each dot represents one gene identified as hub, and the y-axis shows how many times this hub recurs in a sample group.* ***a-h)*** *Plots containing all hubs over all single-sample networks of one subtype or sub-subtype per inference method For each subtype, the subtype-specific hubs are colored in yellow and defined as the hubs (i) occurring in at least 75% of the networks of that subtype and (ii) the hubs selected in (i) that were not overlapping with the selected hubs of the other subtype for the same tissue (lung or brain).*

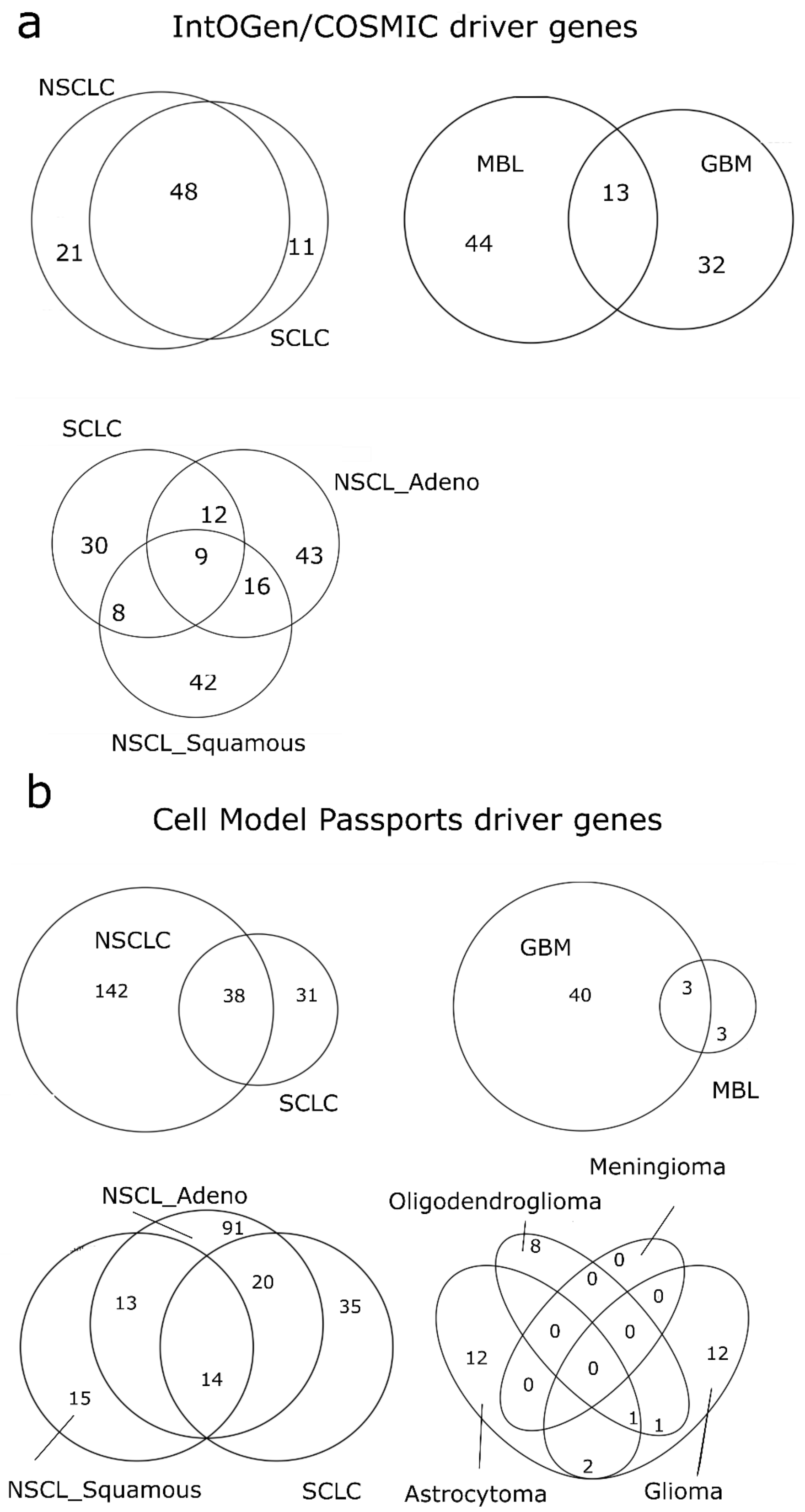

***Supplementary Figure 6. Overlap of known cancer driver genes between different tumor subtypes and sub_subtypes in CCLE from** a) IntOGen/COSMIC and b) Cell Model Passports.*

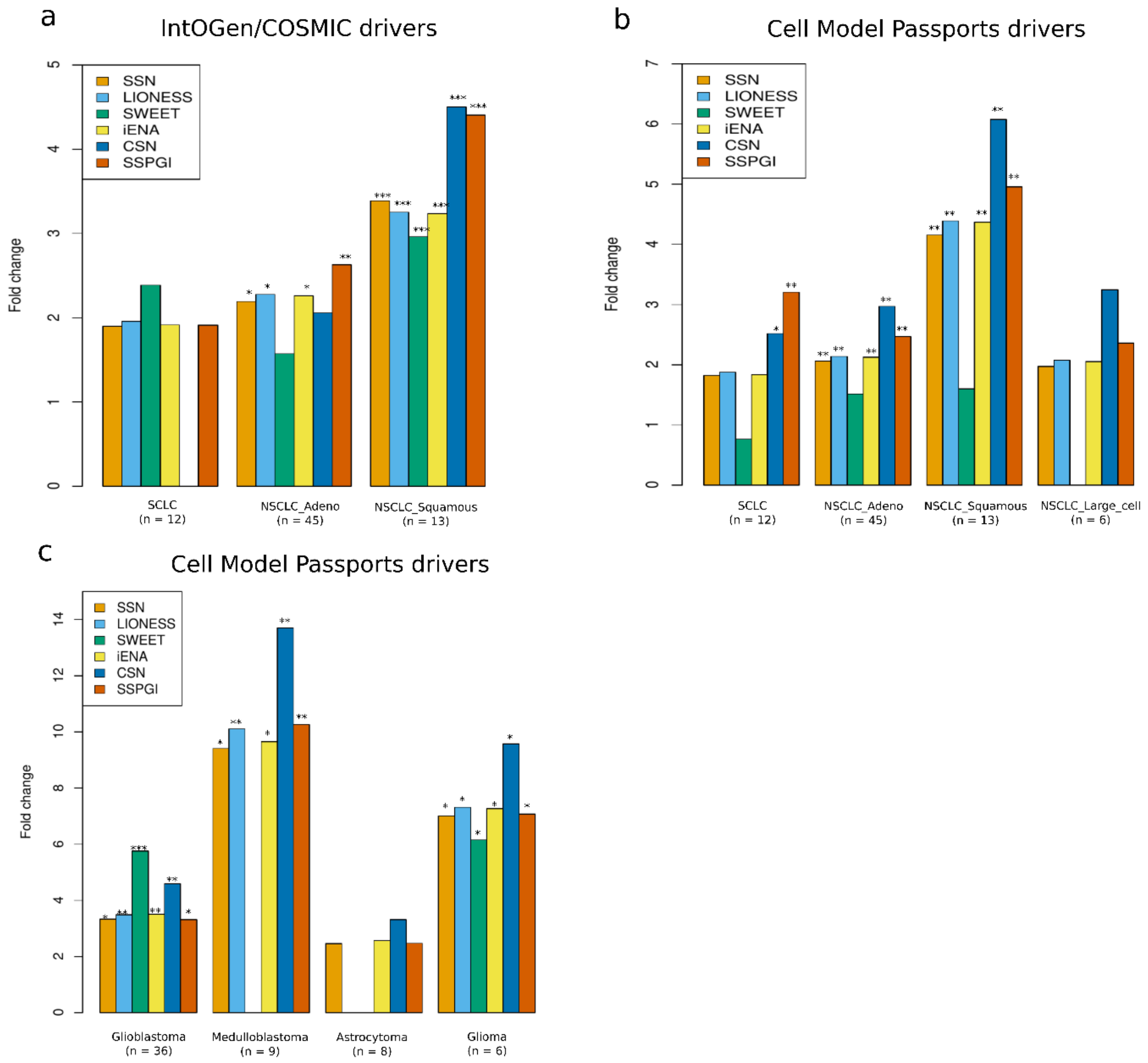

Supplementary Figure 7. Enrichment of known subtype-specific cancer driver genes in hub gene sets upon considering sub-subtypes and more subtypes for lung and brain cancer: a) IntOGen/COSMIC subtype-specific drivers and b) Cell Model Passport drivers for the specific CCLE cell lines for lung cancer, c) Cell Model Passports drivers for the specific CCLE cell lines for brain cancer. *The top 200 most connected nodes in each single sample network were identified as hub genes. Hubs genes were then grouped per sample type and enrichment for known subtype specific cancer driver genes was assessed. (*: $p < 0.05$, **: $p < 0.01$, ***: $p < 0.001$, NSCLC: non-small cell lung cancer, SCLC: small cell lung cancer)*

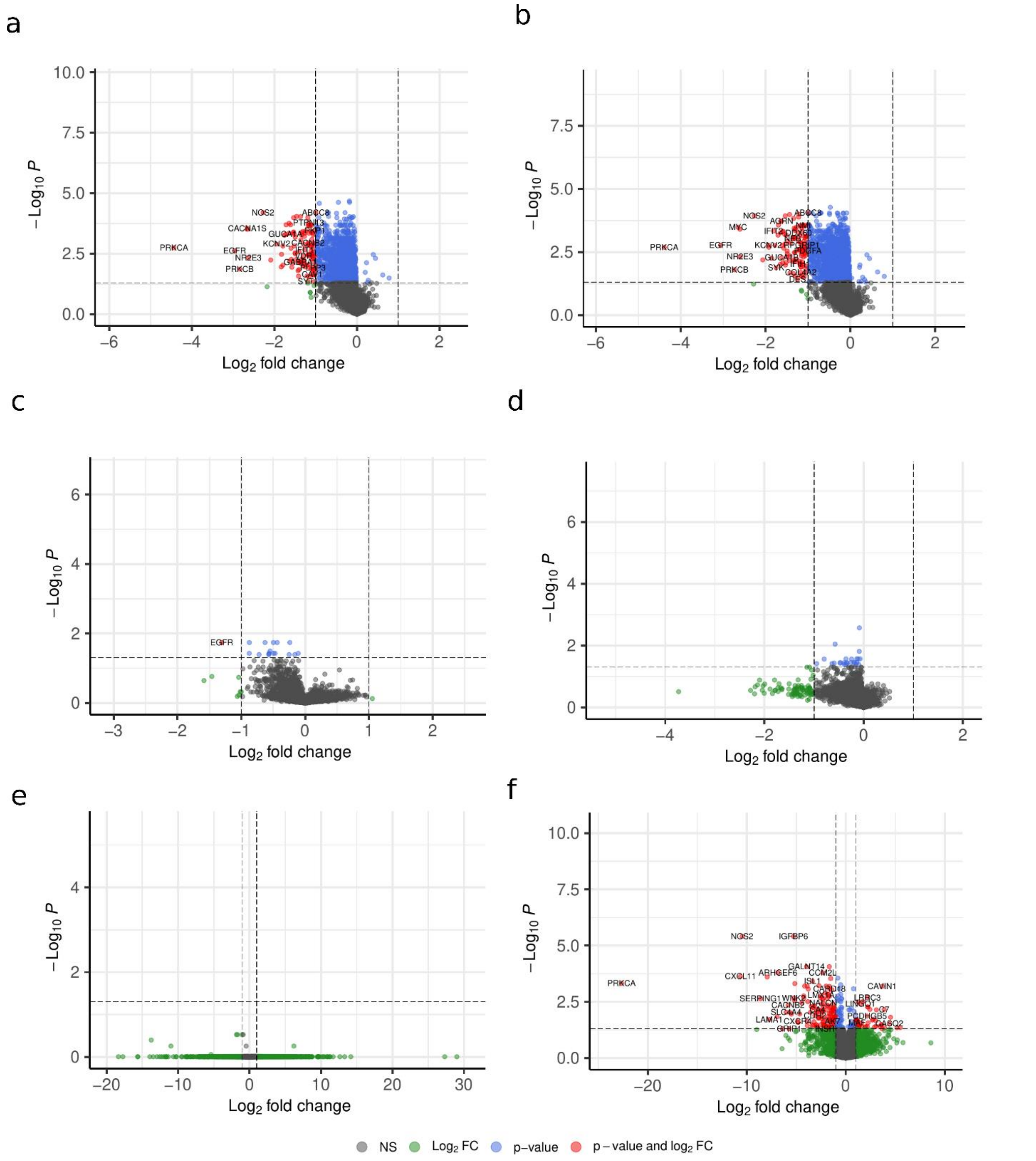

Supplementary Figure 8. Single-sample networks display distinct differential node strength across network types. *The node strength, or sum of absolute edge weights, was calculated for all nodes in top 25k networks constructed by SSN (a), LIONESS (b), SWEET (c), iENA (d), CSN (e) and SSPGI (f). Differentially strong nodes (p-adj < 0.05 & |LFC| >= 1) in glioblastoma versus medulloblastoma (LFC < 0 means lower in glioblastoma than medullablastoma) were identified using linear modelling and an empirical Bayes procedure. (p-adj: adjusted p-value; LFC: log fold change, NS: non-significant)*

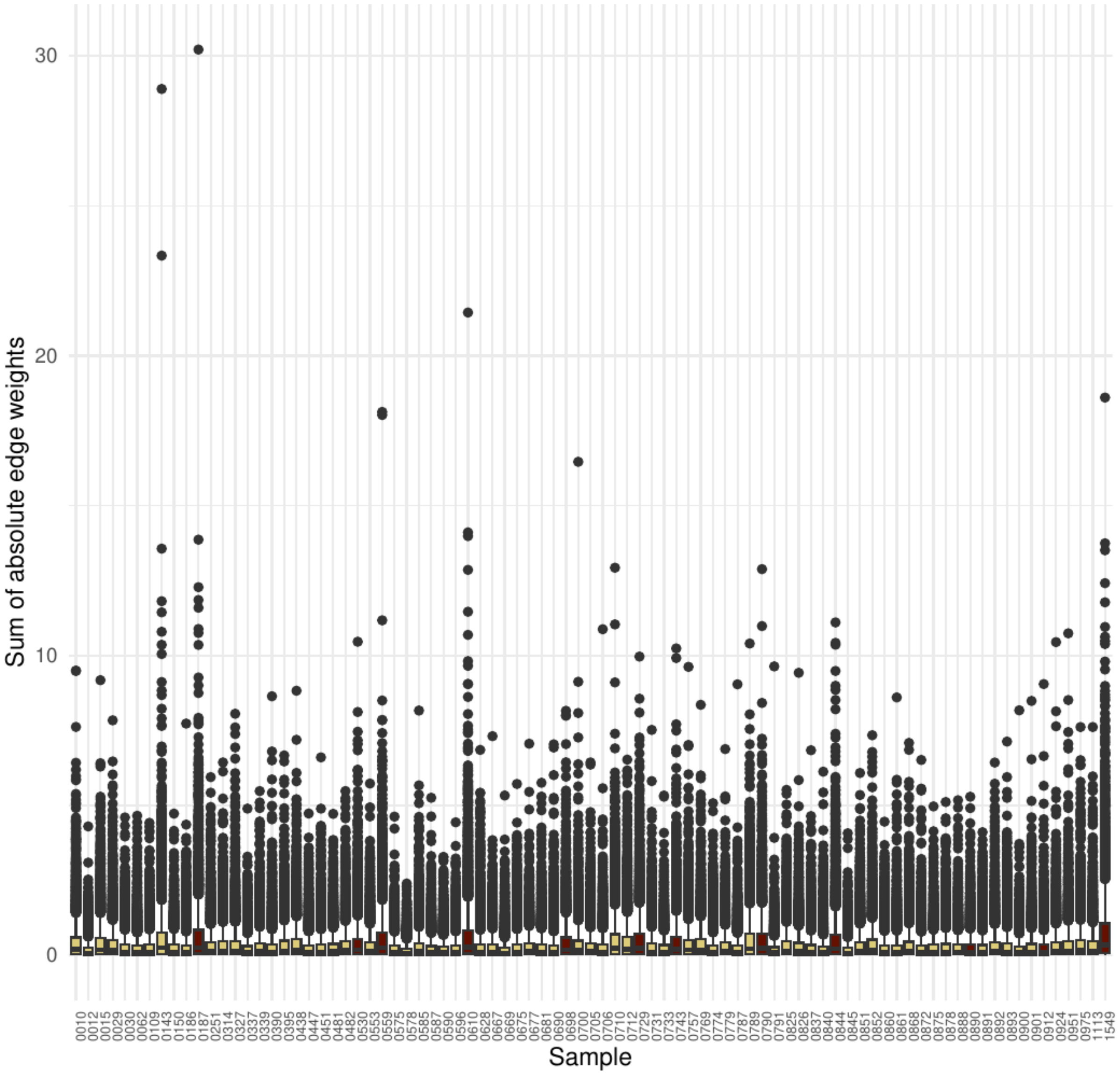

Supplementary Figure 9. SSN lung single-sample networks displayed distinct node strengths between small cell lung carcinomas (n = 12, wine-red) and non-small cell lung carcinoma (n = 73, green-yellow). *The sum of absolute edge weights was calculated for all nodes in top 25k networks constructed by SSN. Boxes represent the 25%-75% interquartile range with the median line, while the outlier points are at least 1.5 times greater than this interquartile range.*

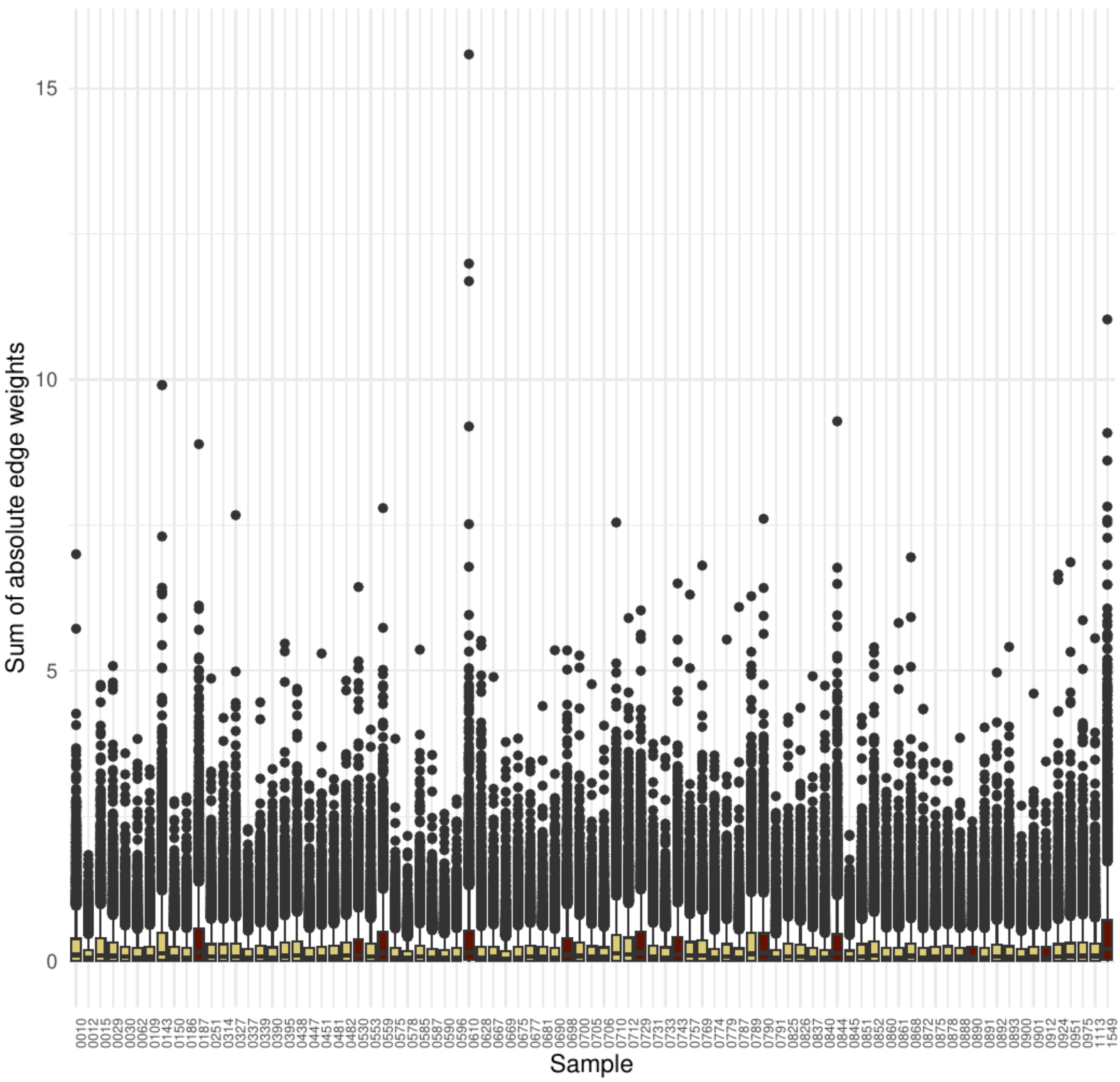

Supplementary Figure 10. LIONESS lung single-sample networks displayed distinct node strengths between small cell lung carcinomas (n = 12, wine-red) and non-small cell lung carcinoma (n = 73, green-yellow). *The sum of absolute edge weights was calculated for all nodes in top 25k networks constructed by SSN. Boxes represent the 25%-75% interquartile range with the median line, while the outlier points are at least 1.5 times greater than this interquartile range.*

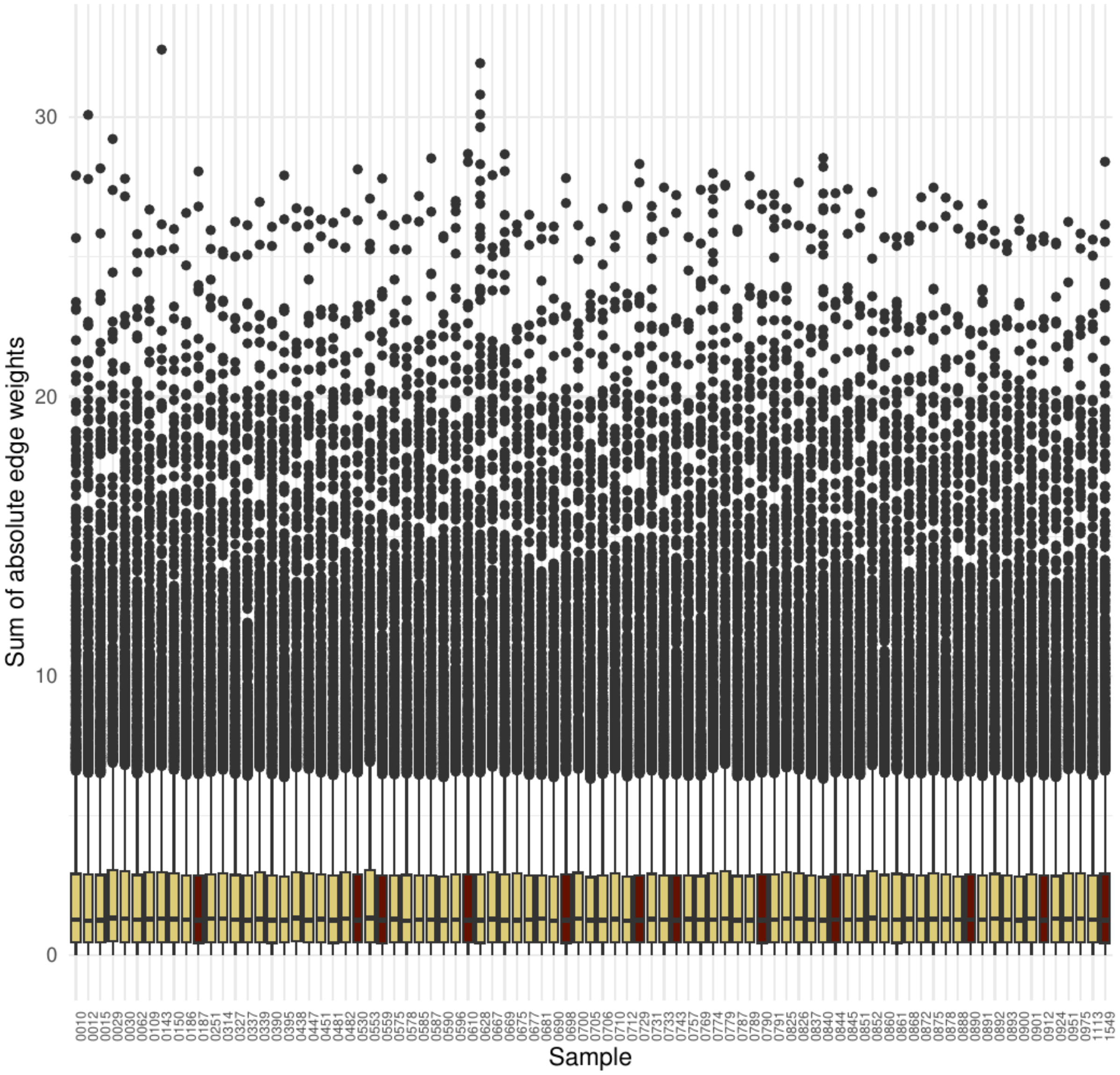

Supplementary Figure 11. SWEET lung single-sample networks did not display distinct node strengths between small cell lung carcinomas (n = 12, wine-red) and non-small cell lung carcinoma (n = 73, green-yellow). *The sum of absolute edge weights was calculated for all nodes in top 25k networks constructed by SWEET. Boxes represent the 25%-75% interquartile range with the median line, while the outlier points are at least 1.5 times greater than this interquartile range.*

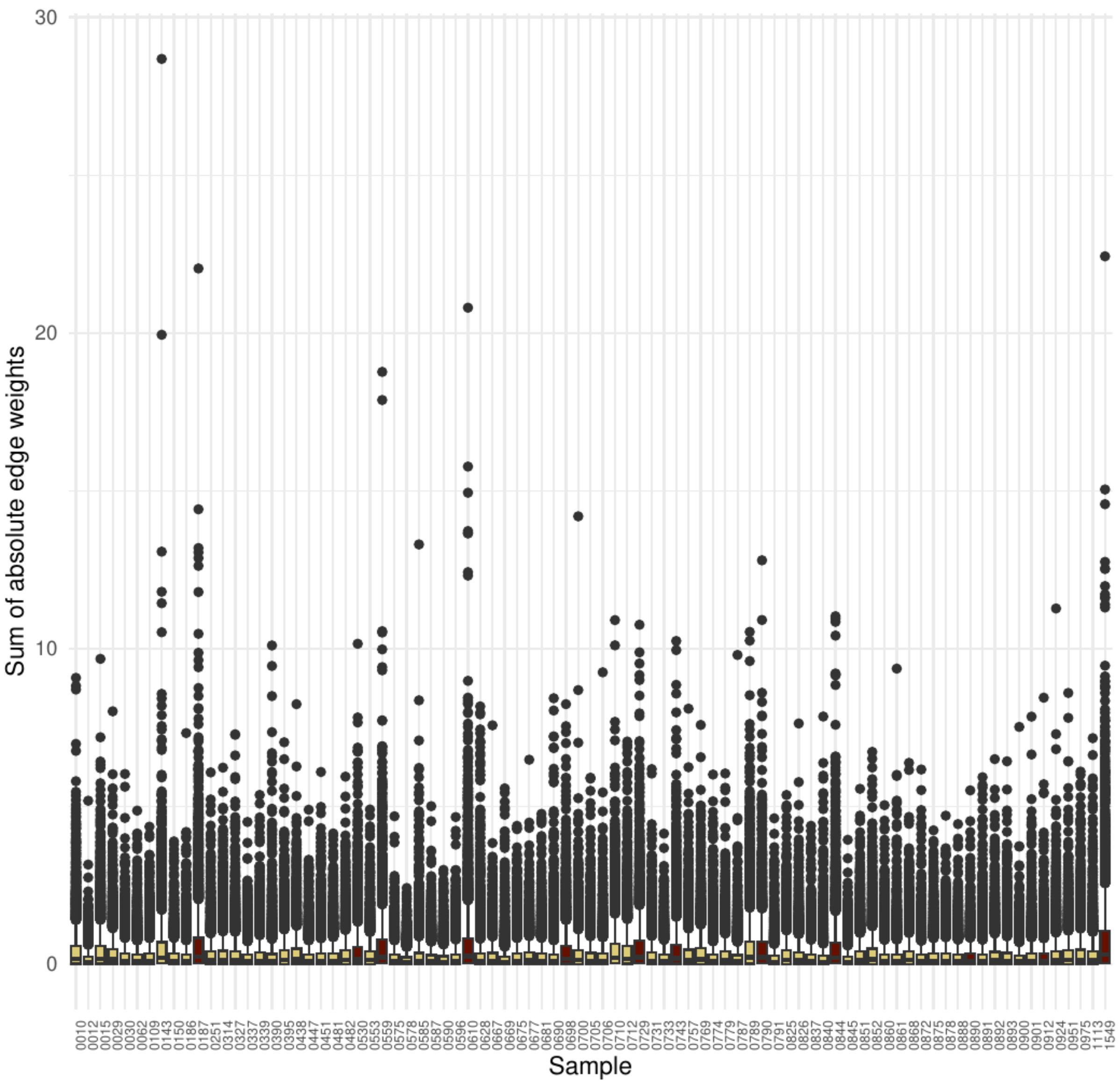

Supplementary Figure 12. iENA lung single-sample networks did not display distinct node strengths between small cell lung carcinomas (n = 12, wine-red) and non-small cell lung carcinoma(n = 73, green-yellow). *The sum of absolute edge weights was calculated for all nodes in top 25k networks constructed by iENA. Boxes represent the 25%-75% interquartile range with the median line, while the outlier points are at least 1.5 times greater than this interquartile range.*

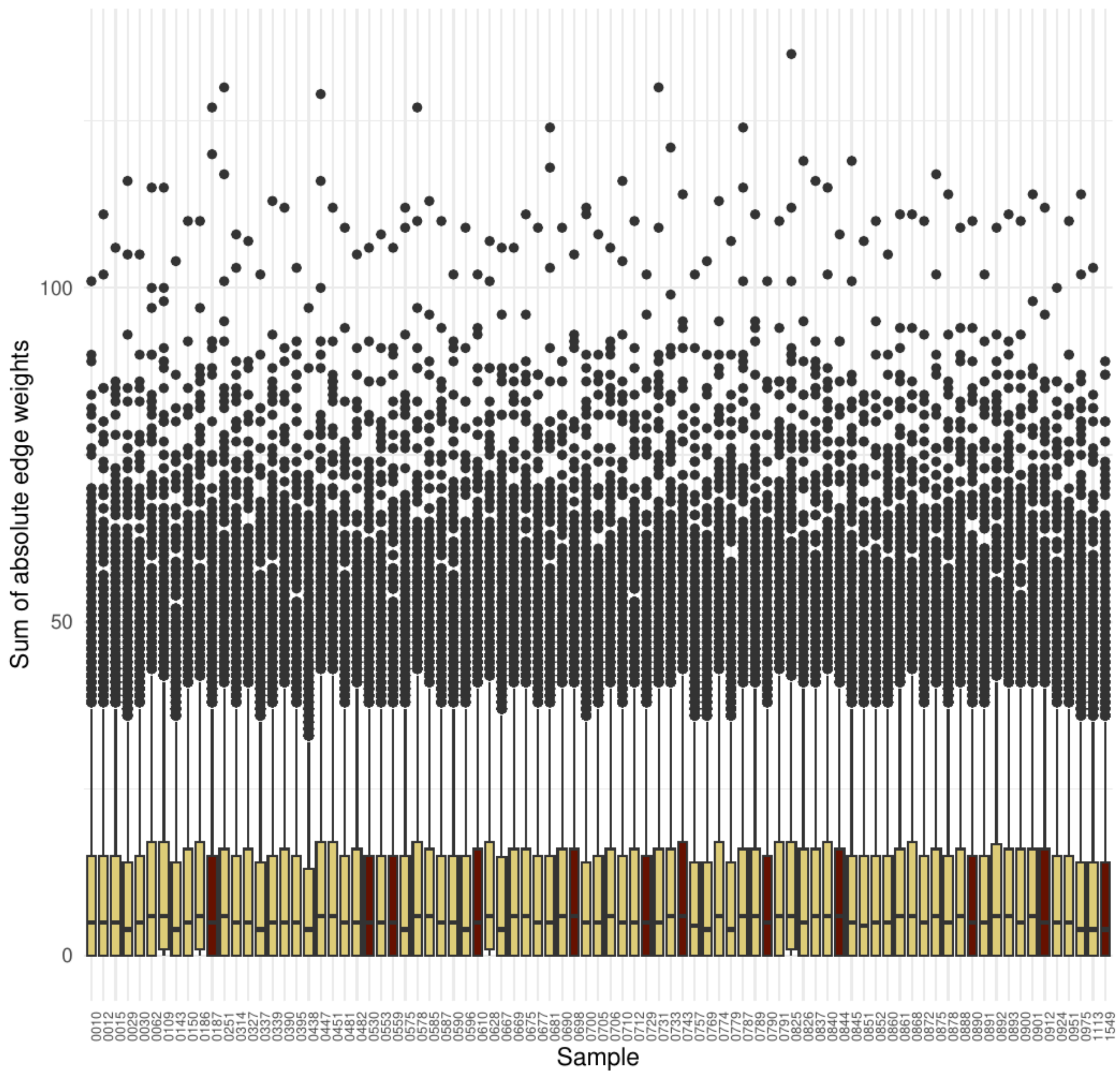

Supplementary Figure 13. CSN lung single-sample networks did not display distinct node strengths between small cell lung carcinomas (n = 12, wine-red) and non-small cell lung carcinoma(n = 73, green-yellow). *The sum of absolute edge weights was calculated for all nodes in top 25k networks constructed by CSN. Boxes represent the 25%-75% interquartile range with the median line, while the outlier points are at least 1.5 times greater than this interquartile range.*

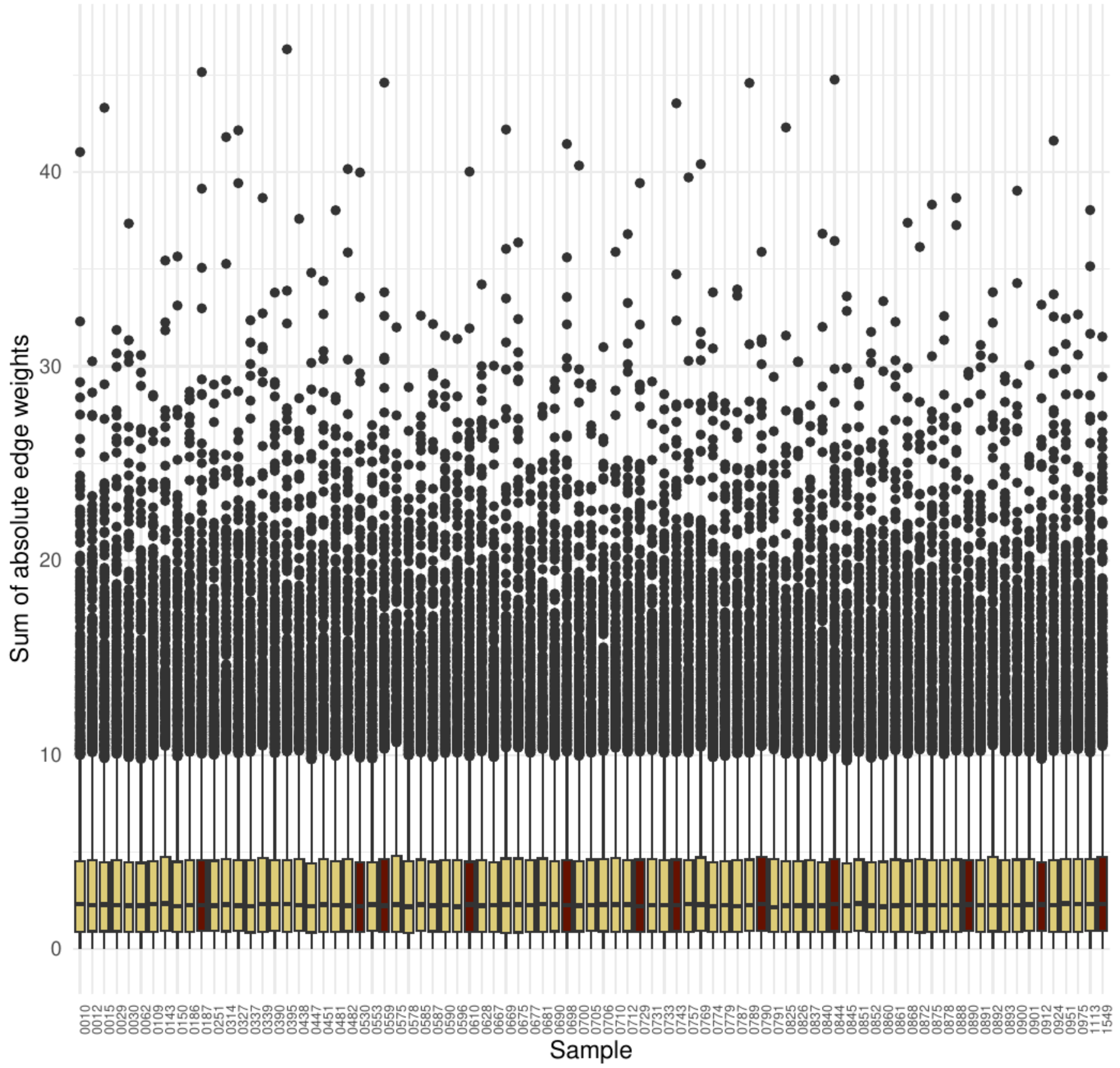

Supplementary Figure 14. SSPGI lung single-sample networks did not display distinct node strengths between small cell lung carcinomas (n = 12, wine-red) and non-small cell lung carcinoma(n = 73, green-yellow). *The sum of absolute edge weights was calculated for all nodes in top 25k networks constructed by SSPGI. Boxes represent the 25%-75% interquartile range with the median line, while the outlier points are at least 1.5 times greater than this interquartile range.*

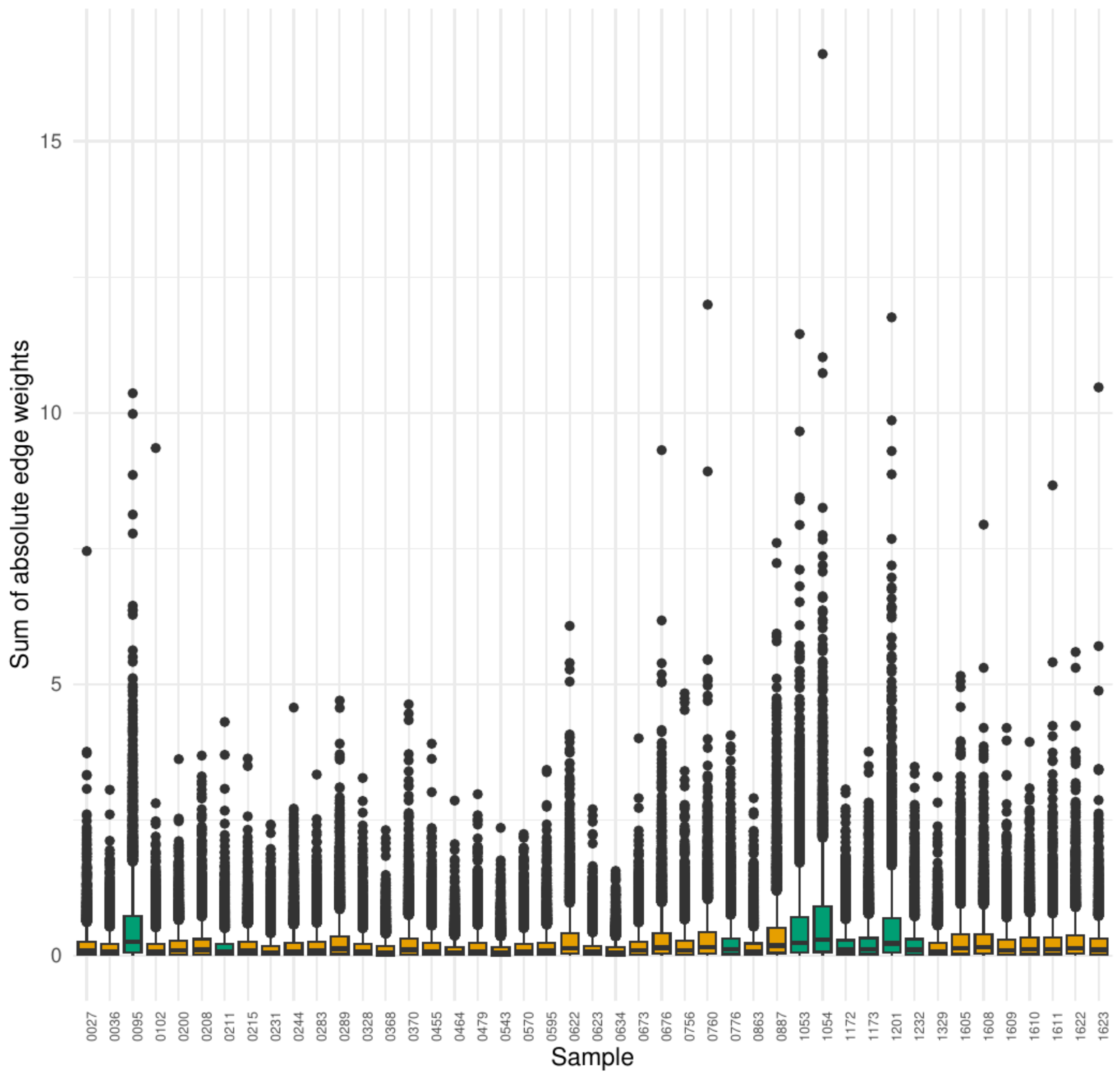

Supplementary Figure 15. SSN brain single-sample networks displayed distinct node strengths between glioblastoma (n = 36, yellow-orange) and medulloblastoma samples (n = 9, green). *The sum of absolute edge weights was calculated for all nodes in top 25k networks constructed by SSN. Boxes represent the 25%-75% interquartile range with the median line, while the outlier points are at least 1.5 times greater than this interquartile range.*

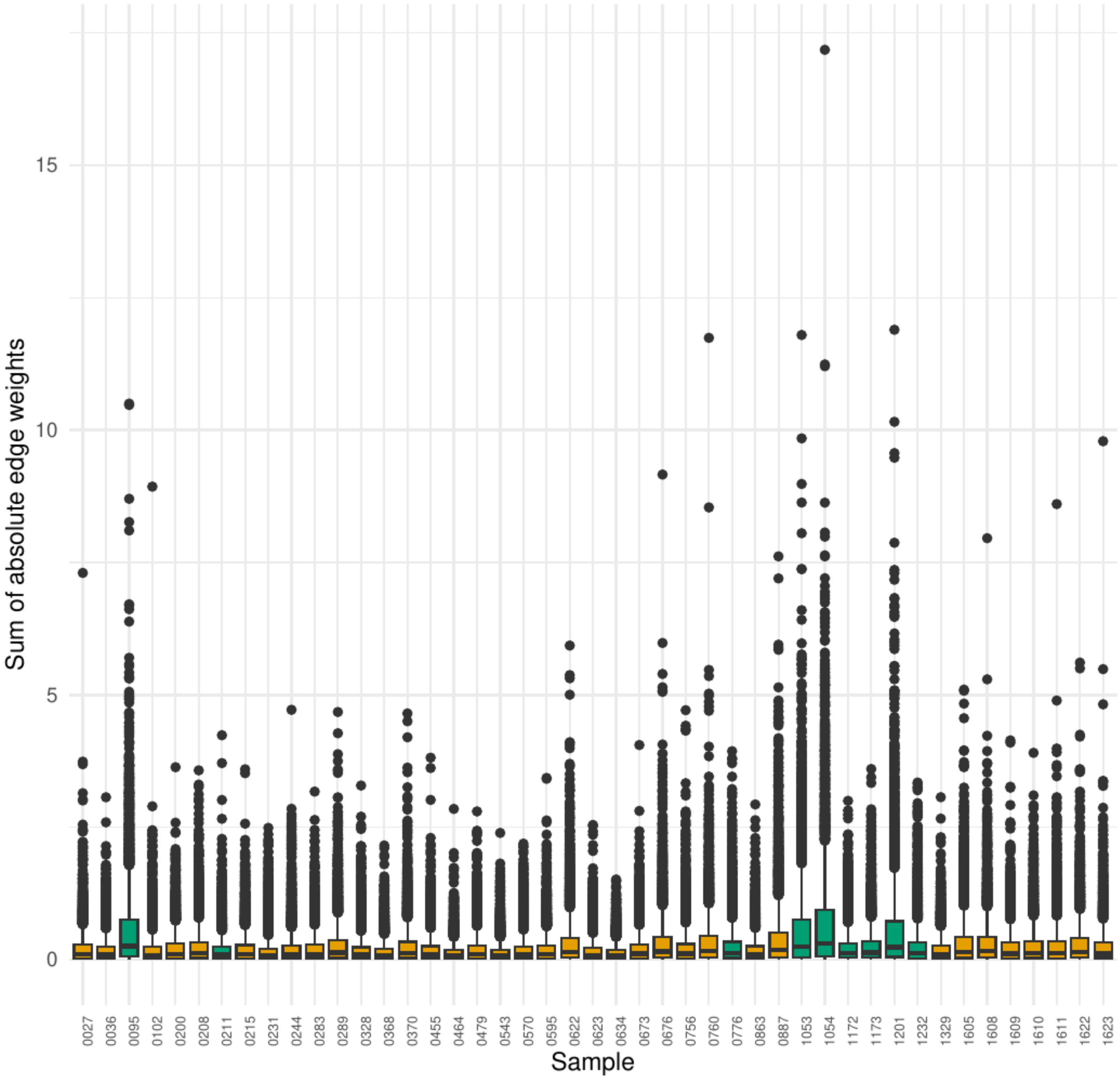

Supplementary Figure 16. LIONESS brain single-sample networks displayed distinct node strengths between glioblastoma (n = 36, yellow-orange) and medulloblastoma samples (n = 9, green). *The sum of absolute edge weights was calculated for all nodes in top 25k networks constructed by LIONESS. Boxes represent the 25%-75% interquartile range with the median line, while the outlier points are at least 1.5 times greater than this interquartile range.*

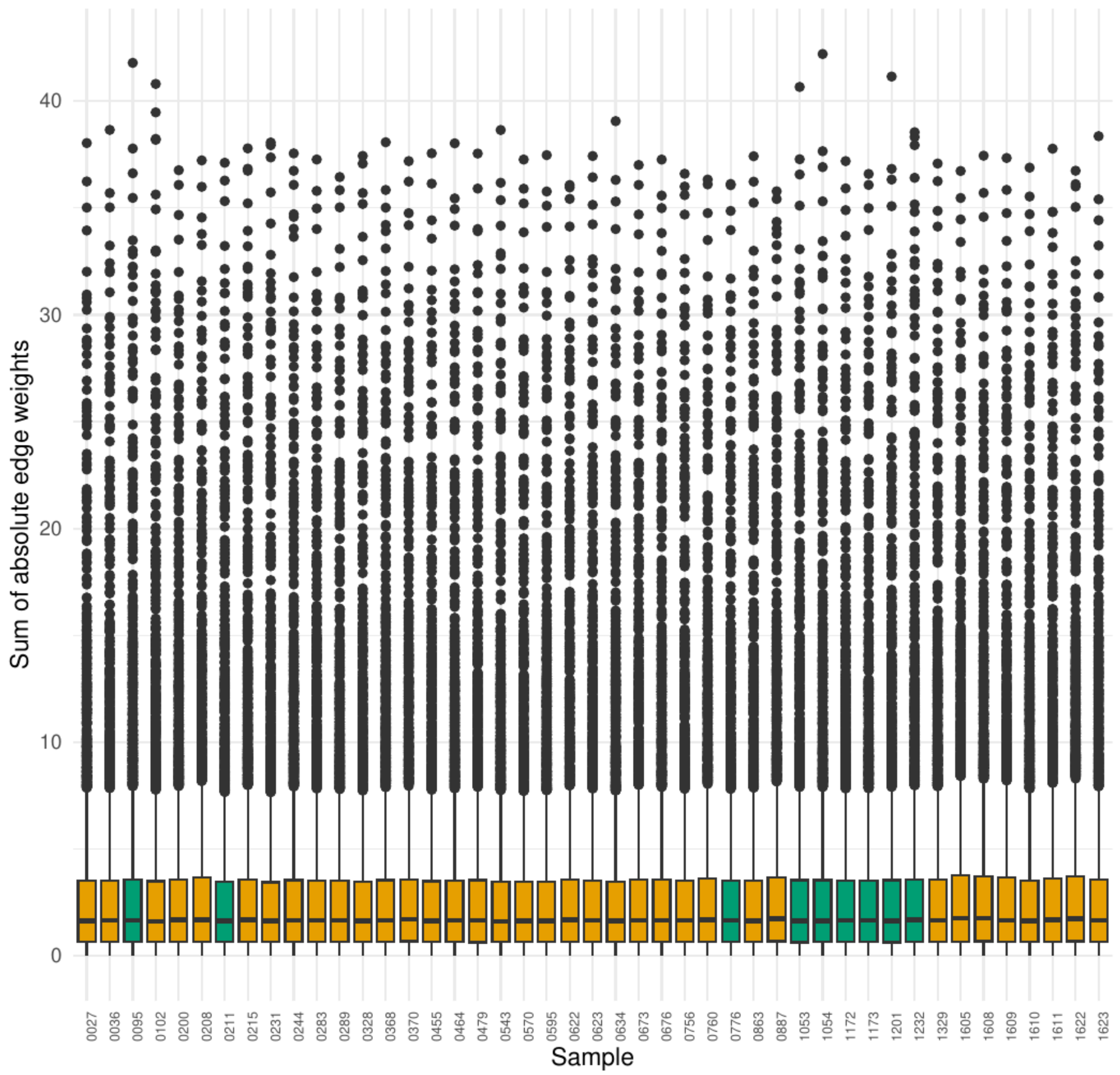

Supplementary Figure 17. SWEET brain single-sample networks did not display distinct node strengths between glioblastoma (n = 36, yellow-orange) and medulloblastoma samples (n = 9, green). *The sum of absolute edge weights was calculated for all nodes in top 25k networks constructed by SWEET. Boxes represent the 25%-75% interquartile range with the median line, while the outlier points are at least 1.5 times greater than this interquartile range.*

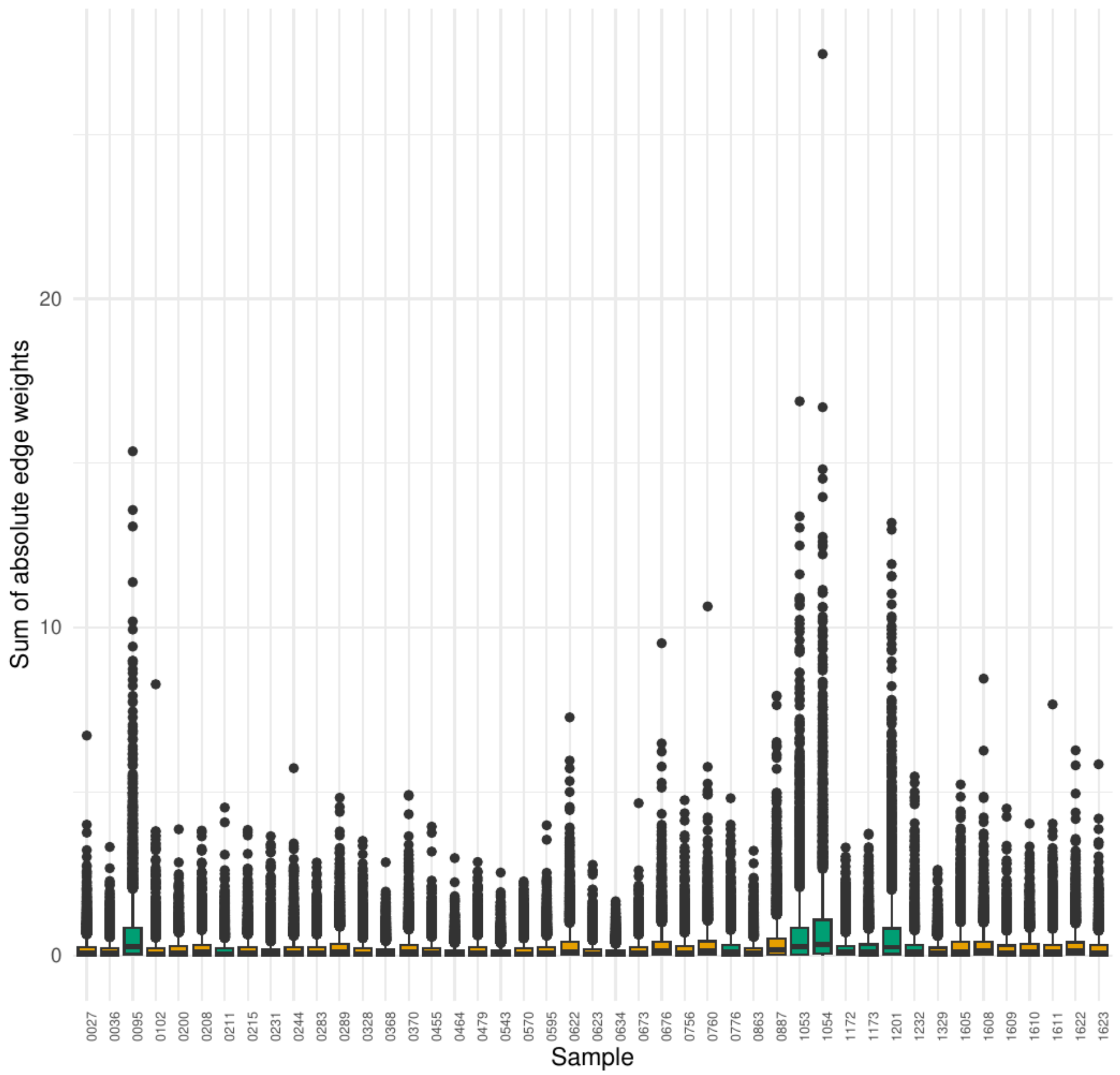

Supplementary Figure 18. iENA brain single-sample networks displayed distinct node strengths between glioblastoma (n = 53, yellow-orange) and medulloblastoma samples (n = 9, green). *The sum of absolute edge weights was calculated for all nodes in top 25k networks constructed by iENA. Boxes represent the 25%-75% interquartile range with the median line, while the outlier points are at least 1.5 times greater than this interquartile range.*

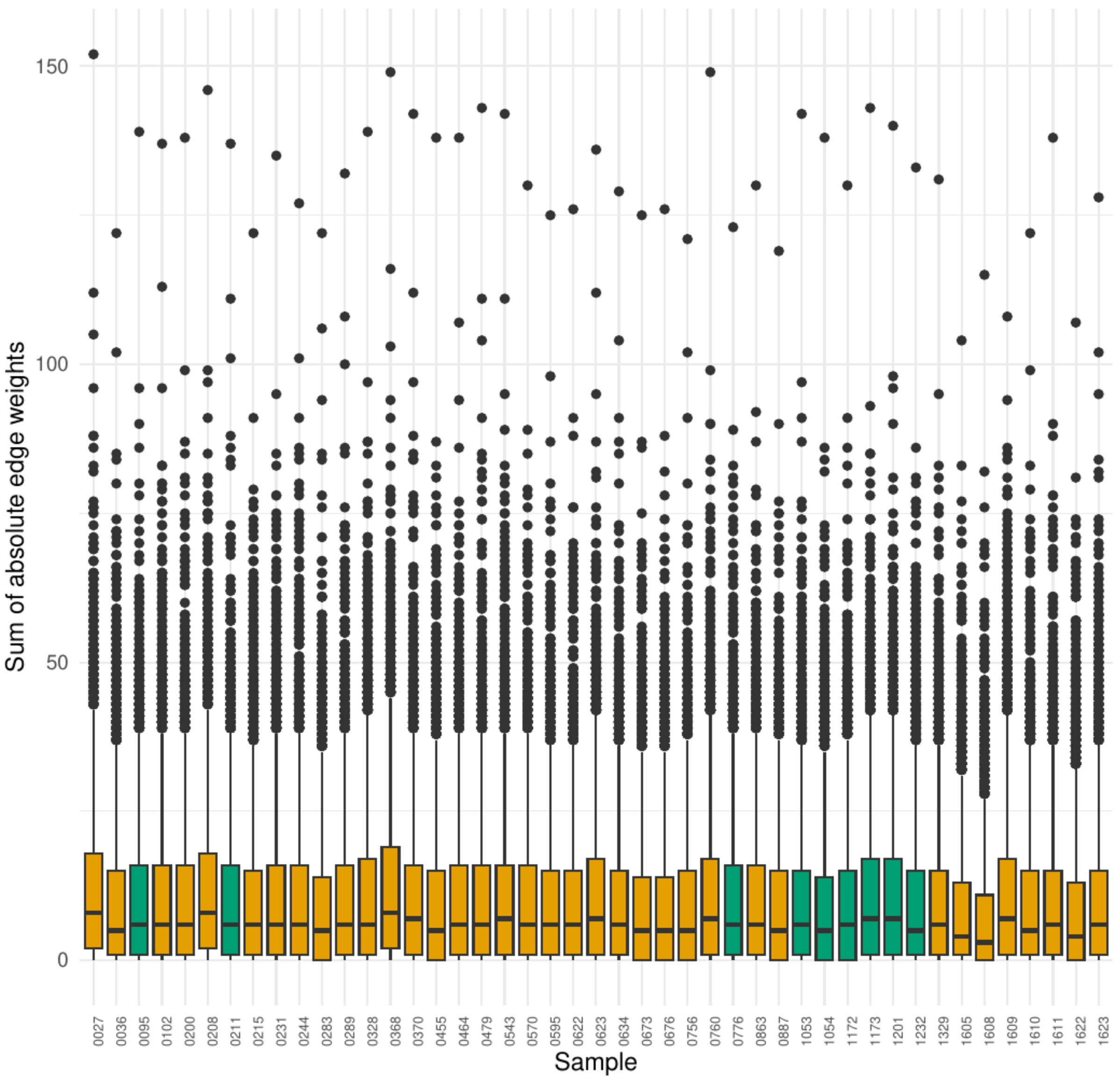

Supplementary Figure 19. CSN brain single-sample networks did not display distinct node strengths between glioblastoma (n = 36, yellow-orange) and medulloblastoma samples (n = 9, green). *The sum of absolute edge weights was calculated for all nodes in top 25k networks constructed by CSN. Boxes represent the 25%-75% interquartile range with the median line, while the outlier points are at least 1.5 times greater than this interquartile range.*

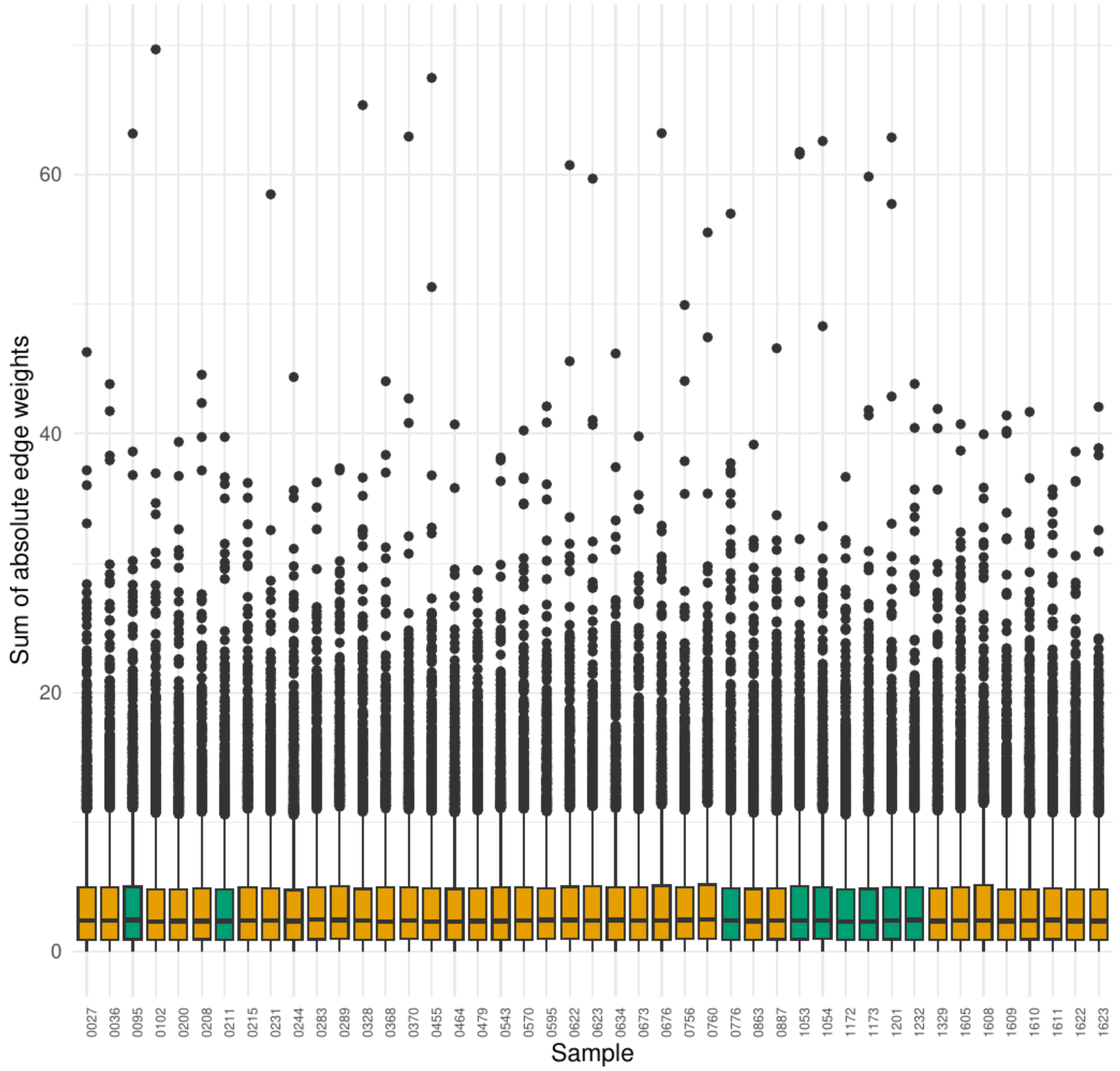

Supplementary Figure 20. SSPGI brain single-sample networks did not display distinct node strengths between glioblastoma (n = 36, yellow-orange) and medulloblastoma samples (n = 9, green). *The sum of absolute edge weights was calculated for all nodes in top 25k networks constructed by SSPGI. Boxes represent the 25%-75% interquartile range with the median line, while the outlier points are at least 1.5 times greater than this interquartile range.*

Supplementary Table 1. Description of network characteristics[36].

CONCEPT	DESCRIPTION
**CLUSTERING COEFFICIENT**	The clustering coefficient measures the degree to which nodes in a network tend to cluster together. It quantifies the likelihood that neighbours of a given node are also connected to each other. High clustering coefficients indicate a highly interconnected network.
**DENSITY**	Density is a measure of how many edges are present in a network compared to the total number of possible edges. It indicates how "full" the network is. High density suggests a denser and more connected network, while low density indicates sparsity.
**NODE BETWEENNESS**	Node betweenness is a centrality measure that quantifies the importance of a node in facilitating communication between other nodes in the network. Nodes with high betweenness are crucial for maintaining network connectivity.
**EDGE BETWEENNESS**	Edge betweenness measures the importance of an edge in connecting different parts of a network. Edges with high betweenness are critical for maintaining efficient communication between nodes and are often considered as potential bottlenecks.
**DIAMETER**	The diameter of a network is the longest shortest path between any pair of nodes in the network. It quantifies the maximum distance between nodes and provides insight into the network's overall size and connectivity.
**CONNECTED COMPONENTS**	Connected components are subgraphs within a network where every node is reachable from every other node within that subgraph. Networks can have one or more connected components, and they reveal the network's structure and isolated subgroups.

Supplementary Table 2. Average network characteristics for single-sample networks of lung samples (n = 86) constructed by different single-sample network inference algorithms. *'# Edges' refers to the total number of edges in the networks after selection for highly variable genes and selection for edges present in the HumanNet network, while '# Edges retained per sample' refers to the average number of non-zero edges in CSN networks, guiding us to select the top 25000 edges per sample in other networks as well. See methods section for more details on how each network characteristic was calculated.*

	**AGGREGATE**	**SSN**	**LIONESS**	**SWEET**	**IENA**	**CSN**	**SSPGI**
**# NODES**	5454	5454	5454	5706	5454	5454	4814
**# EDGES**	53296	53 296	53 296	55 806	53 296	53 296	42 193
**# EDGES RETAINED PER SAMPLE**	25000	25 000	25 000	25 000	25 000	27 813	25 000
**CLUSTERING COEFFICIENT**	0.60	0.28	0.29	0.21	0.30	0.35	0.17
**DENSITY**	0.0028	0.0023	0.0024	0.0018	0.0027	0.0035	0.0023
**NODE BETWEENNESS**	2059.27	7198.60	7043.12	8631.33	6469.36	5556.04	7203.90
**EDGE BETWEENNESS**	0.0002	0.0007	0.0007	0.0006	0.0007	0.0007	0.0007
**DIAMETER**	13.60	10.90	11.58	14.41	10.72	13.77	11.65
**CONNECTED COMPONENTS**	739	19.73	37.97	52.40	20.11	42.27	21.83

Supplementary Table 3. Average network characteristics for single-sample networks of brain samples (n = 67) constructed by different single-sample network inference algorithms. *'# Edges' refers to the total number of edges in the networks after selection for highly variable genes and selection for edges present in the HumanNet network, while '# Edges retained per sample' refers to the average number of non-zero edges in CSN networks, guiding us to select the top 25000 edges per sample in other networks as well. See methods section for more details on how each network characteristic was calculated.*

	AGGREGATE	SSN	LIONESS	SWEET	iENA	CSN	SSPGI
**# NODES**	4741	4741	4741	4936	4741	4741	4686
**# EDGES**	42948	42 948	42 948	45 724	42 948	24 400	42 206
**# EDGES RETAINED PER SAMPLE**	25000	25000	25000	25 000	25000	24399	25000
**CLUSTERING COEFFICIENT**	0.67	0.29	0.29	0.25	0.31	0.34	0.17
**DENSITY**	0.0068	0.0027	0.0024	0.0027	0.0030	0.0037	0.0025
**NODE BETWEENNESS**	1090.21	6509.86	7043.12	7463.56	5937.39	5000.85	6889.49
**EDGE BETWEENNESS**	0.0002	0.0007	0.0007	0.0007	0.0007	0.0008	0.0007
**DIAMETER**	14.37	10.60	11.58	11.13	10.31	13.57	11.25
**CONNECTED COMPONENTS**	576	20.25	37.97	30.79	20.94	45.55	23.93

Supplementary Table 4. Union of hub genes per sample group. *The top 200 most connected nodes were identified as hubs in each single-sample network, after which the union was determined per sample group. (NSCLC = non-small cell lung carcinoma, SCLC = small cell lung carcinoma)*

	SSN	LIONESS	SWEET	iENA	CSN	SSPGI
**SCLC (n = 12)**	624	606	298	619	361	496
**NSCLC (n = 73)**	807	771	437	777	431	665
**NSCLC_Adenocarcinoma (n = 45)**	765	737	400	742	408	638
**NSCLC_Squamous (n = 13)**	656	621	341	624	359	550
**NSCLC_Large_cell (n = 6)**	553	526	309	531	336	462
**Medulloblastoma (n 12)**	504	469	491	491	346	462
**Glioblastoma (n = 36)**	593	566	343	564	430	597
**Astrocytoma (n = 8)**	482	444	269	462	358	479
**Glioma (n = 6)**	451	432	257	435	330	447

Supplementary Table 5. Overview of hub intersection across sample groups and single-sample network inference methods. *The top 200 most connected nodes were identified as hubs in each single-sample network, after which the intersection was determined per sample group. (NSCLC = non-small cell lung carcinoma, SCLC = small cell lung carcinoma)*

	SSN	LIONESS	SWEET	iENA	CSN	SSPGI
**NSCLC (n=73)**	0	0	110	0	97	18
**SCLC (n=12)**	2	3	153	3	125	47
**NSCLC_Adenocarcinoma (n = 45)**	0	0	117	0	101	20
**NSCLC_Squamous (n = 13)**	1	3	135	2	114	34
**NSCLC_Large_cell (n = 6)**	6	8	147	5	123	58
**Medulloblastoma (n 12)**	4	24	167	9	100	50
**Glioblastoma (n = 36)**	0	2	137	0	85	26
**Astrocytoma (n = 8)**	6	27	161	9	104	42
**Glioma (n = 6)**	15	42	168	15	115	54
